# Evaluation of a self-help intervention to promote the health and wellbeing of marginalised people including those living with leprosy in Nepal: a prospective, observational, cluster-based, cohort study with controls

**DOI:** 10.1186/s12889-021-10847-0

**Published:** 2021-05-06

**Authors:** Dilip Shrestha, Indra B. Napit, Subi Ansari, Sopna Mannan Choudhury, Bishnu Dhungana, Paramjit Gill, Frances Griffiths, Holly Gwyther, Deanna Hagge, Shovakhar Kandel, Suraj Puri, Jo Sartori, Samuel Ian Watson, Richard Lilford

**Affiliations:** 1 Anandaban Hospital, The Leprosy Mission Nepal, Tika Bhairav, Lele-9, Lalitpur, P. O. Box 151, Kathmandu, Nepal; 2 Institute of Applied Health Research, College of Medical and Dental Sciences, University of Birmingham, Edgbaston, Birmingham B15 2TT UK; 3 Warwick Centre for Applied Health Research & Delivery (W-CAHRD), Warwick Medical School, University of Warwick, Warwick, UK

**Keywords:** Leprosy, Ulcers, Disability, Low and middle-income countries, Self-care, Self-help groups, Self-management, Economic improvement

## Abstract

**Background:**

People affected by leprosy are at increased risk of ulcers from peripheral nerve damage. This in turn can lead to visible impairments, stigmatisation and economic marginalisation. Health care providers suggest that patients should be empowered to self-manage their condition to improve outcomes and reduce reliance on services. Self-care involves carrying out personal care tasks with the aim of preventing disabilities or preventing further deterioration. Self-help, on the other hand, addresses the wider psychological, social and economic implications of leprosy and incorporates, for example, skills training and microfinance schemes. The aim of this study, known as SHERPA (Self-Help Evaluation for lepRosy and other conditions in NePAl) is to evaluate a service intervention called Integrated Mobilization of People for Active Community Transformation (IMPACT) designed to encourage both self-care and self-help in marginalised people including those affected by leprosy.

**Methods:**

A mixed-method evaluation study in Province 5, Nepal comprising two parts. First, a prospective, cluster-based, non-randomised controlled study to evaluate the effectiveness of self-help groups on ulcer metrics (people affected by leprosy only) and on four generic outcome measures (all participants) - generic health status, wellbeing, social integration and household economic performance. Second, a qualitative study to examine the implementation and fidelity of the intervention.

**Impact:**

This research will provide information on the effectiveness of combined self-help and self-care groups, on quality of life, social integration and economic wellbeing for people living with leprosy, disability or who are socially and economically marginalised in low- and middle- income countries.

**Supplementary Information:**

The online version contains supplementary material available at 10.1186/s12889-021-10847-0.

## Background

### Self-care and self-help

Nerve damage in leprosy leads to a loss of sensation in limbs and around the eyes and inhibits normal sweating which results in the skin becoming dry and fragile. These factors increase the risk of accidental injury and the development of wounds (ulcers) which tend to become infected. Nerve damage can lead to physical disability, either directly or as a result of deep seated infection [1, 2].

In order to promote healing of ulcers and prevent recurrence, people with nerve damage are encouraged to practice self-care. Self-care involves supporting people and teaching them how to look after their hands and feet to prevent and manage ulcers. This incorporates a process of inspecting, soaking, scraping and oiling skin, and dressing wounds. The aim is to keep skin and wounds clean, callus free and well moisturised. Self-care has been found to be successful in preventing the occurrence and recurrence of ulcers [3, 4].

While self-care alleviates the pathophysiological complications of leprosy, the emphasis in self-help is on the socio-economic consequences of the disease. Leprosy is a disease associated with poverty and ultra-poverty (living on less than $0.5 per day) and those who are affected may be even further economically disadvantaged by disability and stigma [5]. Thus, the aim of self-help groups is to assist people to improve their social and economic circumstances by promoting economic self-reliance, social participation and through advocacy [6]. Self-help interventions often include promotion of savings syndicates, provision of seed-corn money, improving skills and know how, and introductions to wider commercial networks.

Self-management interventions may incorporate self-help as in the intervention described in this paper. Organisations concerned with the management of leprosy have widened their remit, and support self-management schemes that include people with disabilities other than leprosy and also people who are simply marginalised with no medical condition.

Two self-management interventions have been previously conducted in Nepal. The CEDAR (Community Empowerment, Development, Disability and Rehabilitation) project [7] ran between 2009 and 2014 in Rautahat (Province 2) and Ramechhap (Province 3), and the PACED (Participatory Action for Community Empowerment and Development) project (unpublished) from 2014 to 2019 in Parsa (Province 2), Chitwan (Province 3) and Makwanpur (Province 3). Although these interventions were viewed as successful, and retrospective (mainly qualitative) evaluations were conducted [7], the results have not been published in the academic literature.

A recent scoping review [8] of community-based self-management interventions among groups that included people affected by leprosy noted 10 studies that reported successful health-related outcomes but none of these were controlled studies. Notwithstanding a recent initiative to promote research in leprosy [9], the evidence base on leprosy-related community interventions is limited and situated primarily in the grey literature. This prospective study thus provides a unique opportunity to develop the evidence on self-care and self-help on clinical, social and economic outcomes in marginalised people.

### Integrated mobilization of people for active community transformation (IMPACT)

The IMPACT programme in Nepal is a five-year self-help and self-care project scheduled to roll out across three districts (Nawalparasi West, Rupandehi and Kapilbastu) in Province 5. Initially designed for people affected by leprosy, the programme has expanded to include other marginalised people, including those with other types of disability (e.g. Lymphatic Filariasis: LF) and people with no disease or disability but who live in extreme poverty. The programme strives to recruit an equal balance of males and females. Participants include people affected by leprosy, people affected by disability or who care for a person with disability and people who are marginalised in other ways. Marginalised people include single women and those with a daily income below $1.9. IMPACT also targets the wider community using advocacy to change negative perceptions of marginalised people and stigmatisation of people with physical or mental illness.

**Fig. 1:**
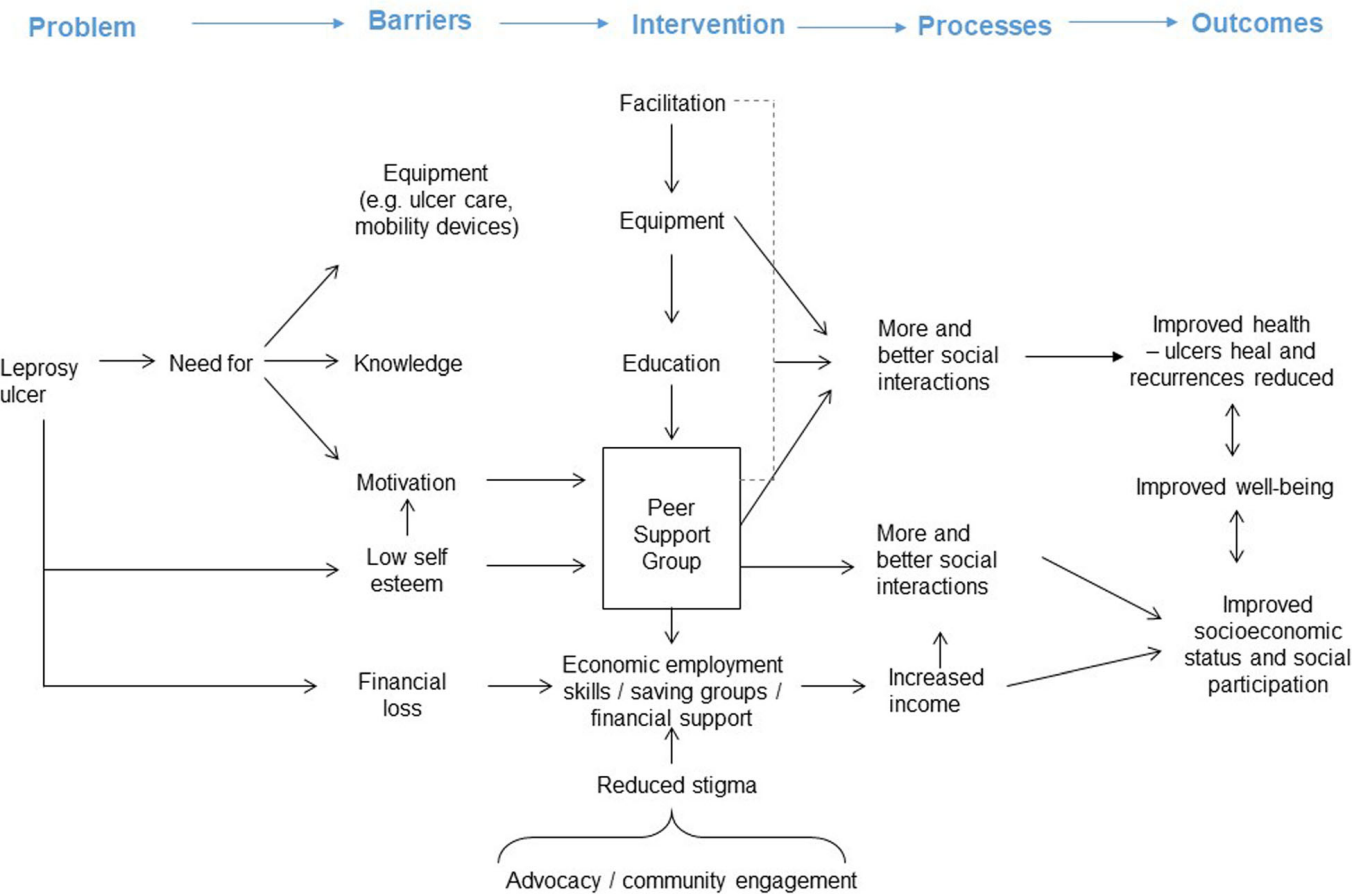
Proposed self-help causal pathway

The aim of the intervention is for individuals to gain knowledge and skills to improve their quality of life. This will be achieved through the formation of 36 self-help groups, which, it is hoped, will become self-sustaining ‘co-operatives’ over time. In summary, individuals will be encouraged to participate in group activities including self-care and income generating activities, to improve their health, wellbeing, social integration and economic status. A self-help causal pathway for the IMPACT intervention is proposed in Fig. 1.

### IMPACT implementation

**Fig. 2:**
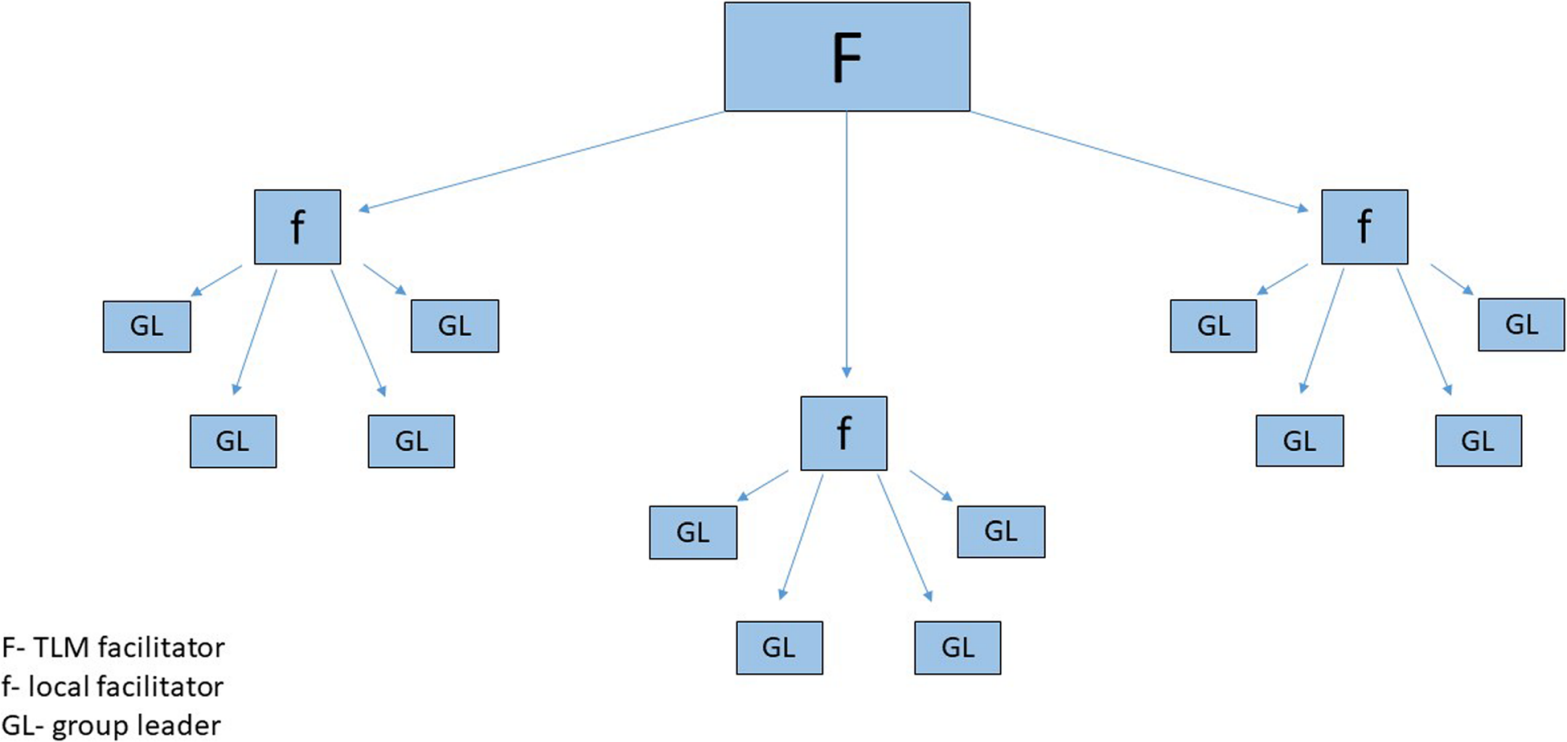
Disbursement of human resources responsible for implementing, mentoring and consolidating the self-care and self-help intervention

The IMPACT intervention is implemented by facilitators employed by The Leprosy Mission (TLM) Nepal. They form a link between the overall governance and management of the programme, and the individual self-help groups in their rural communities. It is the function of the facilitators to bring self-help groups into existence and then mentor their performance. The groups relate to a health outpost in an area of known high prevalence (based on registers of people affected by leprosy and other chronic conditions held at each outpost). About 25 people will be invited to participate in each group and we expect 20 people to ‘sign up’ for the group in each cluster. Facilitators attend group meetings, which will be held fortnightly for three months, and then monthly. The groups elect leaders (chairperson, vice-chairperson, secretary and treasurer), half of whom must be women. These leaders can be changed after two years if necessary. Leaders are given specific training over a three-day period which includes basic accounting, and each group is given assistance to open a bank account. As groups mature, a second ‘layer’ of facilitators are elected to form a ‘bridge’ between the TLM facilitators and the individual groups (Fig. 2).

### IMPACT intervention components

The intervention components are described in Table 1.

**Table 1:** Key components of the IMPACT intervention

Key components	Comments
Group membership	Up to 25 participants, equal numbers of sexes, approximately half of participants affected by leprosy.
Meeting frequency	Sessions lasting approximately 2 h will be held fortnightly for three months and then monthly thereafter.
Facilitation	Three trained facilitators will establish 36 self-help groups. Facilitators will move between groups to provide training in their own speciality area, e.g. budgeting, bookkeeping, wound care. Key tasks include recruitment, encouraging attendance, maintaining registers of group members and activities, monitoring loan repayment, problem solving, engaging in advocacy with influential people in the community.
Peer leadership	The groups will elect four leaders (chairperson, vice-chairperson, secretary and treasurer), half of whom must be women. Leaders can be changed after 2 y if necessary. Leaders will be trained in basic accounting and each group will be given assistance to open a bank account.
Mutual support	Members will be encouraged to share their experiences, support each other and learn from each other.
Encouraging self-care* practices	Facilitators will ensure that group members have appropriate tools and equipment; observe self-care activities and encourage diligence; and encourage early referral to health facilities where necessary.
Tool provision*	Simple tools and equipment will be provided (e.g. mirrors to inspect the plantar surface of the foot, basins for soaking limbs in tap water, crutches, etc.)
Shaping knowledge	Group discussions to include: provision of disability cards, gender violence, civil rights, disaster preparedness. Members will be provided with information and encouraged to adopt healthy behaviours including Water, Sanitation and Hygiene (WASH).
Economic empowerment	Saving credit schemes will be facilitated. Seed money will be provided to establish enterprises. Business awards may be granted after skills training. Groups will be facilitated to become co-operatives that qualify to join the broader co-operative infra-structure in Nepal. Membership of this national, official, collaborative structure confers certain opportunities, such as advice, support and networking opportunities.
Livelihood and skill training	Training will be provided including organic farming, animal husbandry, basic accountancy, sewing, hairdressing.
Advocacy	The facilitators make contact with elected village chiefs, traditional healers, religious leaders and female community health volunteers to advocate for group members.
Ownership	Groups will be autonomous and able to direct and modify their training needs and activities. They will be encouraged to innovate. Past examples include use of games that have been designed for differently abled people and ‘street drama’.

* Only for people affected by leprosy

**Fig. 3:**
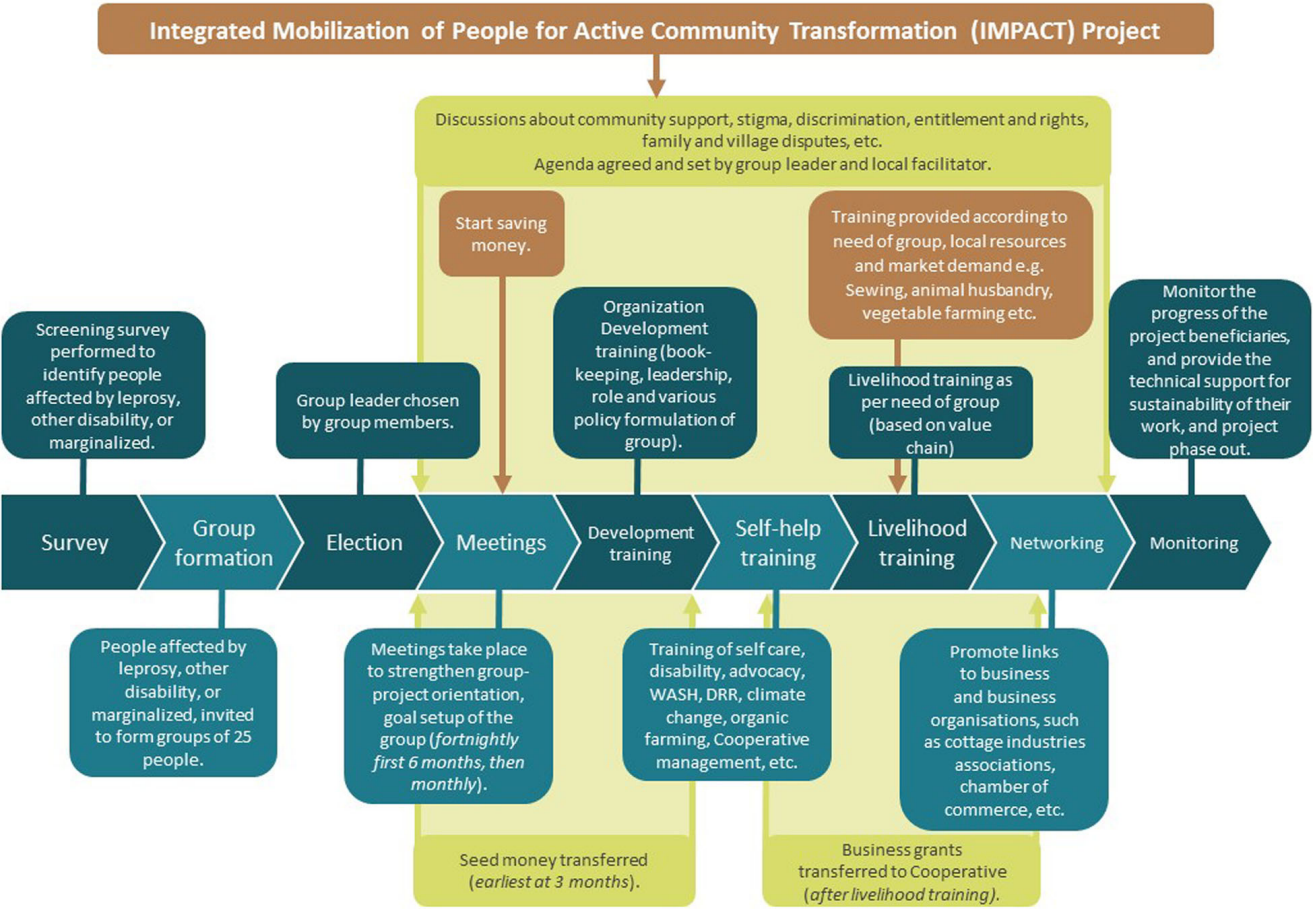
Phasing of the Intervention

These components are not administered simultaneously, but are rolled out over time, as shown in Fig. 3.

The IMPACT intervention was developed by The Leprosy Missions Nepal and Australia, using behavioural science principles. The evaluation team had no part in its development.

The IMPACT intervention is funded by the Australian government at US $150,000 per year for 5 y. This paper describes the independent ‘rapid response’ [10].

## Methods

The SHERPA (Self-Help Evaluation for lepRosy and other conditions in NePAl) study forms part of a wider programme of work under an NIHR Research and Innovation for Global Health Transformation (RIGHT) grant which covers long-term care and prevention of leprosy ulcers in three low-income countries (India, Nepal and Nigeria) and a study of participants with a disease called Buruli ulcer (in Nigeria only). This protocol reports on the evaluation of the IMPACT service intervention in Nepal only.

### Study setting

We will study 18 villages (clusters) of the 36 in the IMPACT self-help intervention in Province 5, Nepal. These villages are selected because they had not already started to implement the IMPACT intervention at the point where ethical clearance for this protocol was obtained. This timing provides an opportunity to conduct a prospective evaluation with baseline, midline and end line observations.

### Study design

The study is a prospective, evaluation of self-help in the selected clusters. We will conduct mixed methods, non-randomised controlled study with observations at baseline, end line and at points in between. People who join the self-help group will form the intervention group (*n* ~ 18*20 = 360). A control group will be obtained from the same clusters as the self-help group (*n* ~ 360). Both groups, intervention and control will be followed up as a cohort. The study will include quantitative observations at baseline and follow up along with qualitative observations with an emphasis on context, the process of implementation and causal mechanisms. Quantitative observations made from all participants (*n* ~ 720) across both groups are of four types: health, wellbeing, social integration and economic wellbeing. These will be made in the self-help groups and control groups. Clinical observations will also be made among the cohort of people affected by leprosy in the self-help group. Since people affected by leprosy are not eligible for the control group, these observations will be made only in the self-help group. Some people affected by leprosy and eligible for the self-help intervention may decline to participate in the self-help group. Any such people will be offered entry into a small, second control group of unknown size described below. The qualitative observations will include observations of both intervention group members, members of the wider community and of senior stakeholders.

### Eligibility criteria

The intervention group (self-help group) will consist of adults at with or at risk of a leprosy ulcer, another chronic disabling condition or who are marginalised by extreme poverty (as defined above). Eligible people will be invited to take part in an IMPACT self-help group. This process is conducted by the IMPACT intervention team independently from the research team who have no part in the process.

**Fig. 4:**
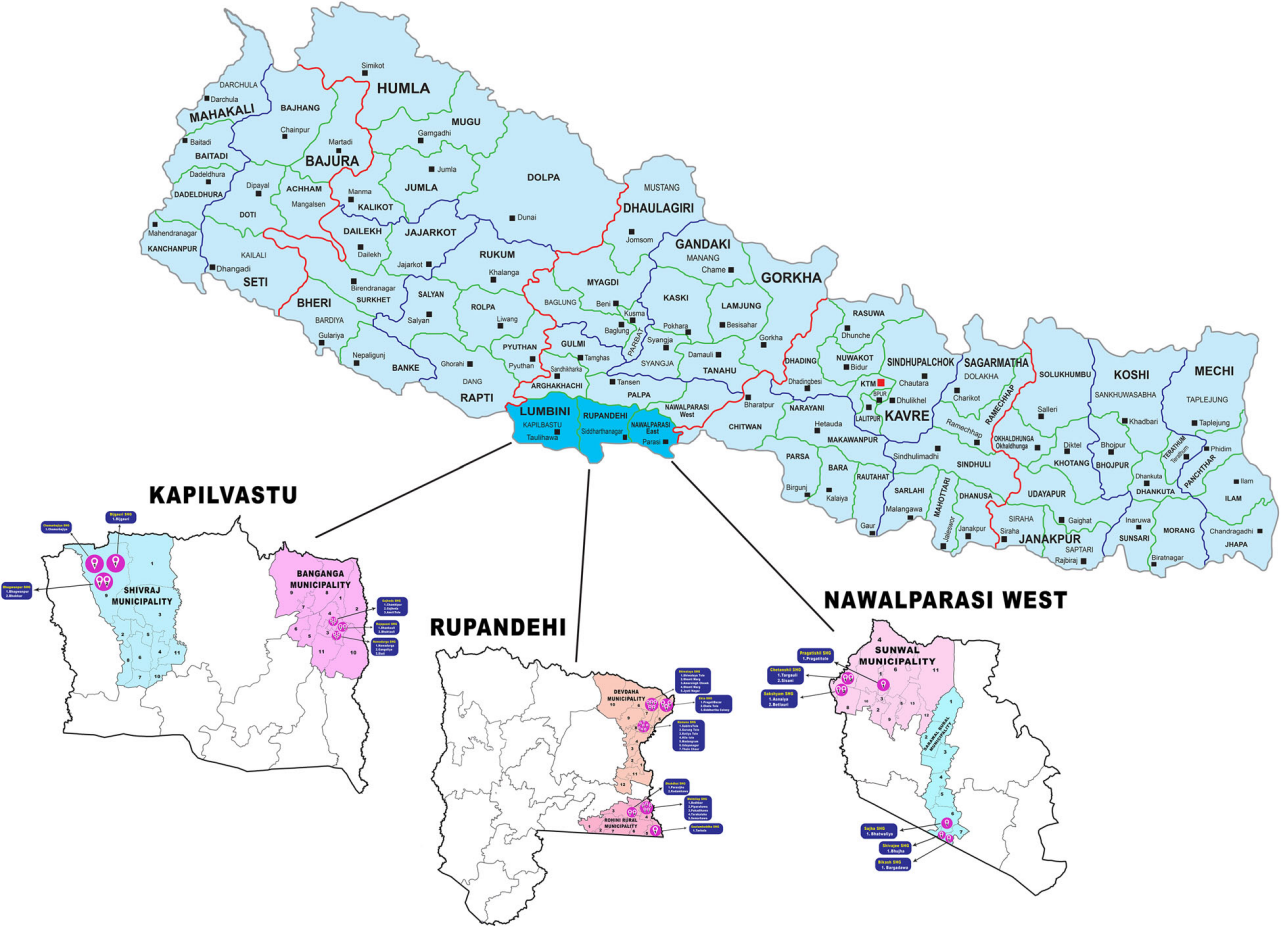
Location of clusters

In Nepal, administrative districts are subdivided into municipalities, then wards, then villages. The IMPACT intervention clusters are created at the level of wards in six municipalities across the three districts (Nawalparasi West, Rupandehi and Kapilbastu) of Province 5. Fig. 4 shows the location of the numbered wards within their respective municipalities and districts. The pink shaded circles in each ward represent the self-help group clusters. Within those clusters, the numbered locations represent adjoining villages, from which prospective self-help group members are recruited.

Figure 4 shows where Provence five is located within Nepal, then the three clusters that comprise the Provence and then the 18 clusters that are taking part in the SHERPA study. Note clusters may include a number of villages from which members of self-help groups (and controls) are selected.

**Fig. 5:**
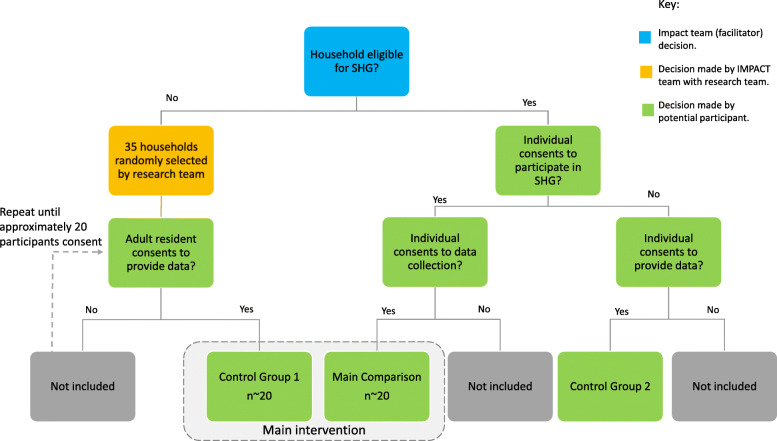
Self-help intervention group decision tree

For comparison purposes, we will generate two additional groups resident in the same clusters as the intervention (self-help group) members. Generating those groups involve collaboration between the IMPACT intervention team and the research group. See Fig. 5.

Control group 1 will be comprised of individual adults (over 18 years of age) who are not eligible to take part in the self-help groups in a particular cluster.

We do not have access to the details of all residents in a cluster, so we cannot directly select people at random to form a counterfactual group to the intervention group. However, we do have access to the details of the households in each cluster. We will therefore start by selecting households at random. The intervention facilitator will obtain a list of all households in the cluster and remove those who are eligible for the self-help intervention from the list. An anonymised list of households will be provided to the study co-ordinator who will randomly select 35 households and place them in a numbered list.

In line with the intervention group, the researcher will identify an adult member of each selected household who is able to answer questions relating to the household’s consumption. The researcher will meet with the identified adults, singly or in groups, to explain the purpose of the study. Each potential participant will be approached individually to seek consent. If the individual declines, then no further action will be taken and the researcher will move on to the next household. The researcher will stop recruiting when approximately 20 people have been recruited from 20 households.

Control group 2 will comprise people who were eligible for the intervention, who declined to participate in the self-help groups, but who nevertheless agree to provide data for the evaluation. It is thus possible, or even likely, that there will be no second control group in some clusters.

### Ethics and consent

Eligible people will be provided with a Participant Information Sheet in local languages. Information will be provided verbally for participants who are non-literate. Written informed consent will be obtained from all participants; or thumb/fingerprints will be requested in lieu of a signature if necessary. Translated consent forms will be back-translated according to the WHO recommendations [11] for quality assurance purposes. Participants will be free to withdraw at any time. Ethics approval for the study has been granted locally in Nepal through the Nepal Health and Research Council (NHRC-approval number 444–2020 P) and by the University of Birmingham Biomedical and Scientific Research Ethics Committee (BSREC).

### Aim of the evaluation

The aim of the SHERPA evaluation is to determine whether the IMPACT intervention achieves its aims to improve health and wellbeing, social functioning and economic status. We hypothesise that:

1. Participants who receive the IMPACT intervention will experience greater improvement over time in the four universal outcome measures (generic health, subjective wellbeing, social participation and economic status) than people in control group 1.
2. People affected by leprosy will experience a lower prevalence and severity of ulcers after the intervention than before.

### Data collection

**Fig. 6:**
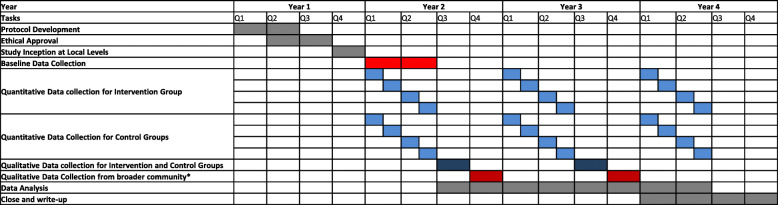
Evaluation Timeline •We have chosen not to specify when baseline data collection will begin due to uncertainty over COVID-related local travel restrictions. •The baseline data collection will last approx. Twenty-four to Twenty-six weeks for all groups. •Quantitative data collection for control and intervention groups will begin together at baseline, and repeat at 12 and 24 months. Quantitative data collection will be staggered, such that for each cluster, data will be collected exactly 12 months apart to avoid seasonal effects. •Qualitative data collection for both the intervention and control groups will also take place 12 months apart, between quantitative data collection periods. •Qualitative data collection from *broader community, e.g. facilitators, community leaders/village chief, healthcare professionals, will follow initial analysis of intervention group interviews, as these will suggest the types of people in the community who are likely to have a mediating effect on the intervention.

Here we describe data collection for intervention group and both control groups. Demographic data will be collected at baseline (T0). Quantitative outcome data (health status, subjective wellbeing, social integration and economic status) will be collected at baseline (T0), 12 months (T1) and 24 months (T2) from intervention and control group participants. The 12-month intervals are designed to mitigate seasonal effects for any given cluster. The evaluation timeline is shown in Fig. 6.

Process evaluation data from qualitative observations for intervention group and control group members will be collected between 7 and 9 and 19–21 months after enrolment (Q3 in Fig. 6). Process evaluation observations from the broader local community will occur between 10 and 12 and 22–24 months after initial enrolment. It is envisaged that group members’ comments will help to determine the most influential community members for interview (refer to eligibility criteria above).

### Demographic data

Demographic data (age and sex), and concurrent diseases will be collected for all consenting intervention and control group participants. Clinical data (e.g., number and size of ulcers) will be collected for people affected by leprosy only.

### Quantitative data

1. *Health, social and economic wellbeing (all participants)*

The four generic outcome measures are:

i. Generic health status (quality of life). Data will be collected using a standardised instrument, the EQ-5D-3L [12]. A country specific digital version will be used. As far as we are aware, there is no valuation tariff in the Nepalese population, although this may change by the time that the analysis starts. Therefore, we propose not to pre-specify the tariff but instead select the tariff at a later date. We note that tariffs are currently available for nearby countries including Sri Lanka [13] and China [14, 15].
ii. Subjective wellbeing data. Data will be collected using a standardised five question instrument [16, 17] designed to capture evaluations of the participant’s level of life satisfaction, an evaluation of personal meaning and happiness, and their affective state.
iii. Social integration. Data will be collected using the validated participation scale (p-scale) [18] which has been used in rehabilitation, stigma reduction and social integration programmes in low- and middle-income countries (LMICs). A Nepali language version is available.
iv. Economic status will be measured by examining total household consumption. A consumption questionnaire has been derived from Sections 2, 5, 6 and 7 of the Nepal: Living Standards Survey (NLSS: 2011/12) [19]; see Additional File 1. The NLSS is a multi-topic household survey conducted by the Central Bureau of Statistics (CBS) of the Government of Nepal in conjunction with the World Bank which in turn is part of the Living Standards Measurement Surveys (LSMS) conducted by the World Bank. We have chosen to measure consumption rather than wealth or income for the following reasons. 1) Measuring income in LMICs is affected by fluctuations due to the informal nature of work and seasonal activity [20], while consumption is more stable over time [20, 21]. Asset (wealth) indices may be more stable [22] but are likely insensitive to change over the short-term observation period of this study.

2. *Clinical Data (people affected by leprosy in intervention group and control group 2)*

Clinical data will be obtained from people affected by leprosy only. Limbs and eyes will be inspected by a researcher in Nepal, and their condition described using a standard form resident on the electronic tablet (see Additional File 2) with information on anaesthesia, ulcers and any visible impairments using the World Health Organisation (WHO) disability grading system [23]. The clinical appearance of any ulcer (e.g. any residual exudate) will be recorded [24, 25].

Ulcer metrics will be based on standardised photographs taken during dressing changes [26]. The measurements will use a photograph taken using the in-built camera in the tablet devices (Samsung Galaxy Tab S6). The photograph will be taken perpendicular to the ulcer. For calibration purposes, a 3 cm size clean paper ruler with date and participant’s identification number will be placed in the photograph frame above or below the ulcer but at the level of the skin. Each photograph will be assessed twice. First, the ulcers will be assessed using the PictZar™ Digital Planimetry Software [17] with an electronic PUSH Tool (National Pressure Injury Advisory Panel (NPIAP) at https://npiap.com/page/PUSHTool) by an independent research fellow in Kathmandu who will be aware of their order in the study timeline. The observer will delineate an area of interest by manually ‘painting’ the ulcer area with colour using a computer mouse. The software will then calculate the ulcer dimensions based on this profile. Second, the photographs will be assessed using the same tool by a research fellow at the University of Birmingham who will be blind to data collection time point. This will ensure that the main outcomes are assessed blind to intervention status and will be done at study end. The total area of all ulcers will be calculated and used as the ‘ulcer area’ metric in the analysis.

### Qualitative data

We will collect data on the implementation of the intervention, examining fidelity and any local adaptations. We will also examine the mechanisms of impact, through participant and facilitator perceptions of the intervention and accounts of how they have acted in response. We will explore facilitators and barriers to intervention success (or not). Finally we will examine the contextual factors (social and environmental) that shape or mediate the intervention in each cluster. These factors will be explored using the methods listed in Table 2.

**Table 2:** Key Components of the IMPACT Process Evaluation

Component	Topic	Method(s)
Implementation	Was the intervention implemented with a high degree of fidelity?	Observations of 18 group meetings (one per cluster) – using template notes Interrogate group attendance logs and activity records (photographed monthly) in each cluster
	Which behaviour change techniques are used?	Observations of group meetings (*n* = 18)
Mechanisms	How do facilitators, group leaders and group members interpret their roles and interact with each other	Observation of group meetings (*n* = 18) Individual interviews with overall facilitator and 3 local facilitators
	To what extent is the intervention sensitive to facilitator/group leader effects?	Observation of group meetings (*n* = 18) and comparison of differences between those groups Individual interviews with overall facilitator and 3 local facilitators Group interviews with a random sample of 9 groups stratified by facilitator
	To what extent (and how) has the IMPACT intervention been effective in facilitating behaviour change amongst participants?	Observations of group meetings (*n* = 18) Group interviews (*n* = 9) stratified by facilitator Individual walking interviews with 2 randomly sampled participants (1 male, 1 female) from 9 groups using photo elicitation
	How, if at all, have self-help group meetings helped participants improve their health, social and economic wellbeing?	Exploration of lived experiences through group interviews (*n* = 9) and individual walking interviews (*n* = 18) using photo elicitation
	What are the experiences of those in the control groups?	Semi-structured individual interviews with 4–5 group members across 9 clusters
Social context (including the social environment)	How does the social context shape the intervention? What is the community perception of people who are differently abled?	Group interviews (*n* = 9) Individual interviews with influential members of the broader local community (details of whom will be derived from earlier interviews and roles/numbers may vary between clusters)
Environmental context	How does the environmental context shape the intervention?	Researcher observations from individual interviews, group interviews and walking interviews on availability of support (e.g. community health workers). Information gathering on proximity to services, water and sewage provision, location of each group, village size (population), crops grown, sources of income, distance from the nearest health facility and town/city using template.

### Power calculation and sample size

As stated, IMPACT will roll out their intervention to 36 clusters and the last 18 to receive the intervention will participate in the evaluation.

IMPACT self-help groups normally consist of 20 participants or more. We will invite all group members to participate in the study. We aim to collect data on at least 20 individuals and so oversampling will allow for drop outs, or any group members who choose not to participate in data collection. We thus aim to recruit intervention and control group 1 members at a ratio of about1:1. See control groups above. Group 2 will be much smaller and we will sample all people in this group (randomly selecting 20 in the unlikely event that this number is exceeded).

We adopt a Bayesian framework for analysis and consider the precision afforded by the sample size from this perspective, i.e. not a power analysis but in terms of the expected width of the 95% credible intervals for the intervention effect. The detailed calculations are laid out in Additional File 3. For our outcomes (expenditure, wellbeing, health status), the smallest effect size for which there is an 80% or greater probability of observing a 95% posterior credible interval that excludes zero is approximately +/− 10% proportionate change in the outcome associated with the intervention.

### Analysis plan

#### Quantitative data analysis

The data will be analysed in a Bayesian hierarchical framework. For the purposes of transparency, we describe our approach to one of the four universal outcomes, that of monthly household consumption in Additional File 3. For the remaining outcomes, we will take a similar approach in terms of hierarchical modelling but will adopt a generalised linear model appropriate to each outcome type.

### Qualitative data analysis

The purpose of the process evaluation is to understand how the intervention is delivered in the local centre, in particular the level of adaptation necessary for the context and environment [27]. We will use framework analysis [28] guided by the Medical Research Council (MRC) Framework for process evaluation of complex interventions [29]. We will code and analyse interview data for:

- Implementation - how the intervention is delivered, and what is delivered in terms of fidelity to intended implementation, dose, adaptations and reach.
- Mechanisms of impact: how do participants respond, what mediates this, and any unanticipated pathways and consequences.
- Context - factors that influence or are affected by the intervention and its outcomes, and that prevent or enable change prompted by the intervention
- Process Outcomes – how the intervention is impacting on the participants’ lived experience and that of the community.

All interviews will be audio recorded, transcribed and translated into English. Observation notes will similarly be translated. The above coding will be applied to all interview data whilst remaining alert to new themes/sub-themes. We will analyse the data by comparing data across sites and between data sources.

Template notes will be collected for each group observed. A summary will be prepared of behavioural interventions delivered, and of how facilitators/group leaders interpret their role, how they interact with the group and how group members respond.

### Data management and monitoring

The University of Birmingham will be the study sponsor. All data generated from this study will be classified according to the University of Birmingham Information Security Framework. All data will be collected and stored electronically. Data will be reported on an electronic Case Report Form (eCRF), and all local and University of Birmingham research staff will be trained to collect data directly onto electronic tablets using REDCap software. Data will be acquired and stored with access restricted by passwords at both the University of Birmingham and the local site in Nepal.

## Discussion

The purpose of the study is to evaluate the effectiveness of the IMPACT self-help group on the health, wealth and wellbeing of marginalised people, including those affected by leprosy. However, there are challenges in the design and approach considered.

The evaluation design has been applied to an existing programme whose roll out has already begun. Moreover, follow up is limited at 24 months because of the duration of the funding envelope. We therefore will not be able to measure how effects may manifest in the longer term. If no effect is observed within the scope of this study, then arguably it is unlikely that one would arise later, especially as we complete our study only shortly before the end of the funded intervention phase. However, we will seek further funds to study sustainability if the findings point towards effectiveness.

It is possible that the observations we make could have an effect, perhaps augmenting the ‘dose’ of any intervention effect. This putative Hawthorne type effect could be observed by making only end-line observations in some clusters. We do not have resources to do so. Furthermore observations will be made in both intervention and control groups, so any reactivity would be equal across groups absent and interaction between the extent of any reactivity and the intervention.

While we have internal controls for health, wellbeing, social and economic data, we obtain clinical data only from people affected by leprosy and people are excluded from control group 1 by design. However, where people affected by leprosy choose not to participate in the intervention, perhaps due to stigma, but nevertheless elect to participate as a member of control group 2, we will be able to collect clinical data. Insofar as these numbers will be small, we may not be able to obtain precise contemporaneously controlled data and will have to rely on before and after comparison. However, we are looking for a much bigger effect on ulcers than on economic change. Moreover, the risk of a temporal trend is greater for the economic and social indicators than for the clinical indicators. Nepal is a developing country so a temporal trend in economic development is to be expected. Absent contemporaneous controls this could lead to a spurious or over-estimated benefit. It was for this reason that we have implemented control groups.

We will use control groups that are ‘internal’ to the clusters where the intervention takes place. We have neither the time nor resources to recruit additional clusters. Also, such clusters may vary systematically from those selected for the intervention. Further, it would be difficult to match participants in control clusters to those participating in the self-help groups in the intervention clusters.

Two issues arise from the use of control groups derived from the same cluster as the intervention; contamination and selection bias:

1. Contamination. The intervention may ‘contaminate’ the control groups leading to an underestimate of treatment effects. To try to mitigate this effect we will not include family members of the participants from the intervention groups. The participants in the intervention group will meet on fortnightly basis whereas participants in control group will not meet in groups. People in the control groups will not meet the facilitators or have access to any of the intervention components such as start out saving money, training or care packages. We believe it to be a reasonable assumption that intra-cluster correlation is principally a result of common exposure to environmental and social factors, rather than interaction between the respondents leading to contamination. In addition, our analyses will allow for flexibility in the specification of random effects to allow shifts in cluster-level variance over time.
2. Selection bias. People who are not disabled are eligible for the self-help because of extreme poverty. We cannot replicate the level of poverty or disability precisely in the counterfactual group. We are thus relying on a difference in the difference when we compare the self-help intervention group with the control group. However, this approach is not assumption free since propensity to change, net of baseline, may differ across groups. We did consider use of the cut off level for the financial definition of extreme poverty with a view to using the threshold as an Instrumental Variable. However, accurate financial data for making such a determination was not available. The clusters are all rural consisting mainly of subsistence farmers and the difference between people in income is small. We will, of course, adjust for such variables as we are able to collect in the analysis and we will ‘triangulate’ our various observations, both qualitative and quantitative [30].

We are proposing a ‘rapid response research’ approach. Rapid response research has produced insights that would otherwise have been missed [31]. However, the timetable is outside the investigators’ control which portends various issues discussed elsewhere [10]. The potential opportunity to evaluate IMPACT prospectively provides a unique opportunity for policy makers and researchers alike. Above all new knowledge will be produced for the benefit of people affected by leprosy specifically and marginalised rural people generally.

A question may be raised with the respect to the value of the second control group. However if the group is very small, as anticipated, then it will not add appreciably to cost. Contrariwise, if it is large it will cost more but provide more precise data in return. Either way, this group will provide valuable insights into participation rates in self-help, reasons for declining and the possible effect of stigma in reducing participation in the very group of people that the self-help intervention was originally intended to benefit.

The evaluation will benefit people with long-term debilitating conditions because the findings will be available to inform implementations around the world. People affected by leprosy (and increasing numbers with diabetes) will benefit from the ulcer specific findings and, along with all people with other marginalizing conditions, from the more general findings regarding the implementation of the theoretically informed IMPACT programme. Our analysis of findings in relation to context will provide information on what works for whom and when. Our emphasis on mediating factors ascertained mostly by qualitative means will inform the questions of why it may work or not, and where and for whom it is likely to work, in the realist tradition [32]. Our use of Bayesian methods reduces the risk that an effective intervention will be declared non-effective on a statistical convention (in direct contradiction of the people who introduced the convention).

On the assumption that even successful interventions can be further improved, our qualitative findings will point the way to future improvements that can then be implemented and evaluated in their turn. Our study will provide a secure base line against which future studies of sustainability can be mounted; lack of proper base-line data from previous studies is an important limitation on studies of the sustainability of previous self-help interventions.

Lastly, we hope that our work in opportunistic or rapid response research will be an inspiration for others who want to evaluate interventions that are likely to be scalable because they have arisen in and from the service, rather than instigated as a research project.

## Supplementary Information

**Additional file 1.** Consumption Questionnaire. Description: A consumption questionnaire derived from Sections 2, 5, 6 and 7 of the Nepal: Living Standards Survey (NLSS: 2011/12) in order to measure economic status.

**Additional file 2.** Clinical Data Collection Form (Patients at Risk of Ulcer Sub-Group). Description: Standard clinical data collection form used by researchers in Nepal to describe condition of patients’ limbs and eyes.

**Additional file 3.** Statistical Model. Description: Calculations regarding the precision of the measurement of the effect size of the intervention.

## Data Availability

Data will be available upon reasonable request.

## Abbreviations

BSREC: University of Birmingham Biomedical and Scientific Research Ethics Committee
CBS: Central Bureau of Statistics
CEDAR: Community Empowerment, Development, Disability and Rehabilitation
eCRF: Electronic Case Report Form
EQ-5D 3 L: 5 Dimensional European Quality of Life Questionnaire
IMPACT: Integrated Mobilization of People for Active Community Transformation
LF: Lymphatic Filariasis
LMIC: Low- and Middle-Income Countries
LSMS: Living Standards Measurement Surveys
MRC: Medical Research Council
NHRC: Nepal Health and Research Council
NIHR: National Institute for Health Research
NLSS: Nepal Living Standards Survey
PACED: Participatory Action for Community Empowerment and Development
PUSH: Electronic Pressure Ulcer Scale for Healing tool
REDCap: Research Electronic Data Capture
RCT: Randomised controlled trials
RIGHT: Research and Innovation for Global Health Transformation (RIGHT)
SHERPA: Self Help Evaluation for lepRosy and other conditions in NePAl
TLM: The Leprosy Mission
WASH: Water, Sanitation and Hygiene
WHO: World Health Organisation
