# Supplementary material for: Evaluation of a self-help intervention to promote the health and wellbeing of marginalised people including those living with leprosy in Nepal: a prospective, observational, cluster-based, cohort study with controls
Source: BMC Public Health. 2021 May 6;21:873. doi: 10.1186/s12889-021-10847-0 (PMC8101219; doi:10.1186/s12889-021-10847-0)
Supplement: Supplementary file 1 — Additional file 1. Consumption Questionnaire. Description: A consumption questionnaire derived from Sections 2, 5, 6 and 7 of the Nepal: Living Standards Survey (NLSS: 2011/12) in order to measure economic status. [file 12889_2021_10847_MOESM1_ESM.docx]

**Consumption questionnaire**

Participant ID

**Section 2 Housing**

**Part B: Housing Expenses**

Is this dwelling yours? Yes


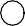

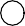


No

What is your present occupancy status? Renter

Provided free of charge by relatives, landlord or employer


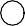

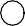


Squatting Other


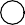

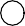


What is the rent per month? (Cash plus value of in-kind payments)

**Part C: Utilities and amenities**

Where does your drinking water come from? Piped Water Supply Covered Well


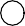

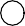

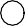

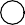

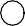

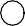

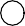


Hand Pump/ tube well Open Well

Spring Water River

Other Source


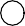

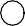


Do you have water piped into your house? Yes No

How many hours per WEEK does your household have tap water? (HRS/WEEK)

How much did you pay for water over the last 12

months? (EXCLUDE WATER USED FOR IRRIGATION) WRITE ZERO IF NOTHING (Rupees)

Are you connected to a sanitary system for liquid Underground drains

wastes? Open drains


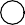

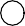

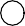

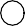


Soak pit No

How does your household dispose of its garbage mainly? Collected by garbage truck

Private/community collector Dumped


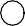

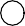

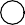

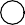

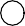

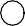


Burned/buried

Dumped and used for fertilizer Other

How much do you pay for garbage disposal over the last

12 months? WRITE ZERO IF NOTHING (RUPEES)

What type of toilet is used by your household? Household flush (connected to municipal sewer)


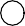

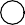

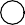

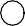

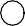


Household flush (connected to septic tank) Household non-flush

Communal latrine No toilet

What is the main source of lighting for your dwelling? Electricity

Solar Biogas Kerosene Other


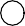

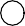

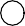

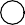

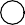


Do you have a joint or individual electric meter? Individual Joint

No meter


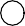

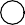

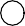


How much did you spend on electricity over the past 12

months? (RUPEES) WRITE ZERO IF NOTHING

In the past 7 days, how many hours (total) did your

household NOT have electricity? (HRS)

Which of the following facilities are there in your Telephone


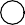

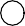

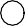

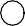

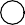

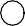


dwelling unit? Smart Mobile Phone

Older (analogue/non-smart) mobile phone Cable T.V./Dish T.V./Net T.V

Cable Internet None

How much did you pay for using those facilities listed

above over the last 12 months?(Rupees)

What kind of fuel is most often used by your household Firewood for cooking? Dung

Leaves/ rubbish/straw/thatch Cylinder gas


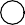

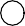

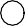

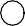

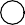

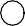

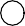


Kerosene Bio-gas Other

What type of stove does your household mainly use for Open fireplace cooking? Mud

Smokeless oven Kerosene stove Gas stove


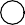

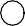

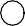

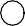

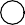

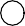


Other

Did your household use any firewood over the past 12 Yes months? No


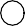

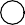


Did your household collect any firewood in the past 12 Yes months? No

How many bharis/carts/kg of firewood did you collect

during the past 12 months?


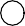

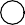


How long does it take to collect one bhari/cart/kg of

firewood? (HRS/Minutes)

Where did you MAINLY collect the firewood? Own land


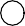

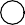

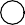

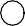


Community managed forest Government forest

Other

How much did you pay for each bhari/cart/kg? (Rupees) WRITE ZERO IF NOTHING

Did you collect fodder for your livestock over the Yes

past 12 months? No


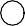

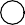


Where did you mainly collect the fodder? Own land

Community managed forest Government forest


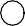

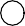

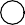

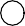


Other

**Section 5 Food expenses and home production**

Have you consumed GRAINS AND CEREALS during the past Yes 12 months? No


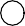

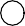


(e.g. fine rice; coarse rice; beaten, flattened rice; maize; maize flour; wheat flour; millet; other grains/cereals)

How many days has the household consumed Grains and Cereals during the past 7 days?

(* IF NONE, WRITE ZERO )

How much Grains and Cereals did your household consume during the past 7 days? (In KGS)

What is the value of this quantity of Grains and

Cereals consumed during the past 7 days? (In Rupees)

MAIN SOURCE for Grains and Cereals Home Production Purchases

In-Kind


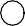

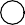

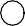


Have you consumed Pulses and Lentils during the past Yes 12 months? No

(e.g. Black Gram (Mas); Lentil (Musuro ); Red Gram; Horse Gram (Chana ); Other pulses (Green Gram, Masyang , Vetch, Khesari , etc.); Beans)


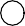

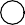


How many days has the household consumed Pulses and

Lentils during the past 7 days? (* IF NONE, WRITE ZERO )

How much pulses and lentils did your household consume

during the past 7 days? (KGS)

What is the value of this quantity of pulses and

lentils consumed during the past 7 days?(Rupees)

MAIN SOURCE for pulses and lentils. Home Production Purchases

In-Kind


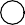

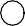

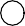


Have you consumed Eggs and Milk Products during the Yes past 12 months? No


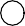

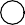


(PRODUCTS (e.g. eggs; milk; condensed milk; baby milk/powder milk; curd/whey; other milk products like cheese, paneer)

How many days has the household consumed eggs and milk products during the past 7 days?

(* IF NONE, WRITE ZERO )

What is the value of the quantity of eggs and milk

products consumed during the past 7 days?(In Rupees)


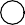

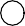

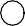


MAIN SOURCE for eggs and milk products. Home Production Food Purchases In-Kind

Have you consumed cooking oil during the past 12 Yes

months? No


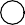

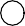


(e.g. ghee; vegetable oil; mustard oil; other oil (soya, sunflower, corn etc.))

How many days has the household consumed cooking oil

during the past 7 days? (* IF NONE, WRITE ZERO )

How much cooking oil did your household consume during

the past 7 days?(In Litres)

What is the value of this quantity of cooking oil

consumed during the past 7 days?(In Rupees)

MAIN SOURCE for cooking oil. Home Production Purchases

In-Kind


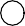

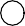

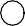


Have you consumed vegetables during the past 12 Yes

months? No


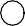

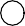


(e.g. potatoes; colocassia; onions; cauliflower/cabbage; tomatoes; green leafy vegetables; pointed gourd; bitter gourd; other vegetables)

How many days has the household consumed vegetables

during the past 7 days? (* IF NONE, WRITE ZERO )

How much vegetables did your household consume during

the past 7 days?(In KGS)

What is the value of this quantity of vegetables

consumed during the past 7 days?(In Rupees)

Main Source For Vegetables. Home Production Purchases

In-Kind


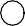

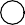

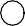


Have you consumed fruits and nuts during the past 12 Yes months? No


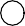

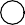


((e.g. bananas; citrus fruits (orange, lemon, lime, sweet orange, pomelo etc.); mangoes; pineapple; papaya; other fruits (grape, pomegranate); dried fruits (walnut, coconut etc.))

How many days has the household consumed fruits and nuts during the past 7 days

(* IF NONE, WRITE ZERO )

How much fruits and nuts did your household consume during the past 7 days?(In KGS)

What is the value of this quantity of fruits and nuts

consumed during the past 7 days?(In Rupees)

Main Source For Fruits and Nuts Home Production Purchases

In-Kind


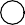

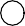


Have you consumed fish and meat during the past 12 Yes

months? No

(e.g. fish; mutton; buffalo meat; chicken; other meats (pig, boar, duck, etc.))

How many days has the household consumed fish and meat

during the past 7 days ? (* IF NONE, WRITE ZERO )

How much fish and meat did your household consumed

during the past 7 days?(In KGS)

What is the value of this quantity of fish and meat

consumed during the past 7 days?(In Rupees)

Main Source for Fish and Meat Home Production Purchases

In-Kind

Have you consumed spices and condiments during the Yes past 12 months? No

(e.g. salt; cumin seed/black pepper; turmeric; ginger/garlic; chilies; other spices and condiments (coriander, nutmeg, clove etc.))

How many days has the household consumed Spices and

Condiments during the past 7 days? (* IF NONE, WRITE ZERO )

How much spices and condiments did your household

consume during the past 7 days?

What is the value of this quantity of spices and

condiment consumed during the past 7 days?(In rupees)

Main Source for Spices and Condiments Home Production Purchases

In-Kind

Have you consumed sweets and confectionary during the Yes past 12 months? No

(e.g. sugar; gur (sakhar); sweets (mithai); other sweets (sugar candy; chocolate etc.))

How many days has the household consumed sweets and confectionary during the past 7 days?

(* IF NONE, WRITE ZERO )

How much sweets and confectionary did your household consume during the past 7 days?

What is the value of this quantity of sweets and

confectionary consumed during the past 7 days?(In Rupees)

Main Source for Sweets and Confectionary. Home Production Purchases

In-Kind

Have you consumed non alcoholic beverages during the Yes past 12 months? No

(e.g. tea (dried leaves); coffee (ground, instant); fruit juices/ Carbonated drinks (Coca cola, Pepsi cola, etc.); Other non-alcoholic drinks (Mineral water, Sarbat , etc.))

How many days has the household consumed non alcoholic

beverages during the past 7 days? (* IF NONE, WRITE ZERO )

How much non alcoholic beverages did your household

consume during the past 7 days?(Litres)

What is the value of this quantity of non alcoholic

beverages consumed during the past 7 days?(In Rupees)

Main Source for Non alcoholic Beverages Home Production Purchases

In-Kind

Have you consumed alcoholic beverages during the past Yes 12 months? No

(e.g. wine; gin; whiskey; beer/jandh; other alcoholic drinks (tadi etc.))

How many days has the household consumed alcoholic

beverages during the past 7 days? (* IF NONE, WRITE ZERO )

How much alcoholic beverages did your household

consume during the past 7 days?(Litres)

What is the value of this quantity of alcoholic

beverages consumed during the past 7 days?(In Rupees)

Main Source For Alcoholic Beverages Home Production Purchase

In_kind

Have you consumed tobacco and tobacco products during Yes the past 12 months? No

(e.g. Cigarettes; bidis; tobacco; other (jarda, khaini, betel nut))

How many days has the household consumed tobacco and

tobacco products during the past 7 days?

How much tobacco and tobacco products did your

household consume during the past 7 days?(Packets)

What is the value of this quantity of tobacco and

tobacco products consumed during the past 7 days?(In rupees)

Main source for Tobacco and Tobacco Products. Home Production Purchases

In-Kind

Have you consumed miscellaneous food products during Yes the past 12 months? No

(e.g. Meals taken outside home; Bread/ biscuit / Noodles; Misc. other food expenditures)

How many days has the household consumed miscellaneous

food products during the past 7 days? (* IF NONE, WRITE ZERO )

How much miscellaneous food products did your

household consume during the past 7 days?

What is the value of this quantity of miscellaneous

food products consumed during the past 7 days?(In Rupees)

Main Source for Miscellaneous food Products. Home Production Purchases

In-Kind

AVERAGE WEEKLY EXPENDITURE ON FOOD (In Rupees)

AVERAGE WEEKLY VALUE OF HOME PRODUCED FOOD (In Rupees)

AVERAGE WEEKLY VALUE OF FOOD RECEIVED IN KIND (In

Rupees)

AVERAGE WEEKLY TOTAL (Rupees)

AVERAGE MONTHLY EXPENDITURE ON FOOD (In Rupees)

AVERAGE MONTHLY VALUE OF HOME PRODUCED FOOD (In

Rupees)

AVERAGE MONTHLY VALUE OF FOOD RECEIVED IN KIND (In

Rupees)

AVERAGE MONTHLY TOTAL (Rupees)

**SECTION-6 Non-food expenditures and inventory of durable goods**

**PART: A Frequent Non-Food Expenditures**

Were wood (bundle wood, logwood, sawdust) purchased or Yes received in-kind over the past 12 months? No

What is the money value of the amount of wood (bundle

wood, logwood, sawdust) purchased or received in-kind by your household during the past 30 days? (Rupees)

What is the money value of the amount of wood (bundle

wood, logwood, sawdust) purchased or received in-kind by your household during the past 12 months? (Rupees)

Were kerosene oil purchased or received in-kind over Yes

the past 12 months? No

What is the money value of the kerosene oil purchased

or received in-kind by your household during the past 30 days? (Rupees)

What is the money value of the kerosene oil purchased

or received in-kind by your household during the past 12 months? (Rupees)

Were coal/charcoal purchased or received in-kind over Yes the past 12 months? No

What is the money value of the coal/charcoal purchased

or received in-kind by your household during the past 30 days? (Rupees)

What is the money value of the coal/charcoal purchased

or received in-kind by your household during the past 12 months? (Rupees)

Were cylinder gas (LPG) purchased or received in-kind Yes over the past 12 months? No

What is the money value of the cylinder gas (LPG)

purchased or received in-kind by your household during the past 30 days? (Rupees)

What is the money value of the cylinder gas (LPG)

purchased or received in-kind by your household during the past 12 months? (Rupees)

Were matches, candles, lighters, lanterns, etc. Yes purchased or received in-kind over the past 12 months? No

What is the money value of the matches, candles,

lighters, lanterns, etc. purchased or received in-kind by your household during the past 30 days? (Rupees)

What is the money value of the matches, candles,

lighters, lanterns, etc. purchased or received in-kind by your household during the past 12 months? (Rupees)

Were readymade clothing and apparel purchased or Yes

received in-kind over the past 12 months? No

What is the money value of the readymade clothing and

apparel purchased or received in-kind by your household during the past 30 days? (Rupees)

What is the money value of the readymade clothing and

apparel purchased or received in-kind by your household during the past 12 months? (Rupees)

Were cloth, wool, yarn, and thread for making clothes Yes

and sweaters purchased or received in-kind over the No past 12 months?

What is the money value of the cloth, wool, yarn, and

thread for making clothes and sweaters purchased or received in-kind by your household during the past 30

days? (Rupees)

What is the money value of the cloth, wool, yarn, and

thread for making clothes and sweaters purchased or received in-kind by your household during the past 12

months? (Rupees)

Were tailoring expenses done in the past 12 months? Yes No

What is the money value of the tailoring expenses

spent by your household during the past 30 days? (Rupees)

What is the money value of the tailoring expenses

spent by your household during the past 12 months? (Rupees)

Were footwear (shoes, slippers, sandals, etc.) Yes purchased or received in-kind over the past 12 months? No

What is the money value of the footwear (shoes,

slippers, sandals, etc.) purchased or received in-kind by your household during the past 30 days? (Rupees)

What is the money value of the footwear (shoes,

slippers, sandals, etc.) purchased or received in-kind by your household during the past 12 months? (Rupees)

Were toilet soap purchased or received in-kind over Yes

the past 12 months? No

What is the money value of the toilet soap purchased

or received in-kind by your household during the past 30 days? (Rupees)

What is the money value of the toilet soap purchased

or received in-kind by your household during the past 12 months? (Rupees)

Were toothpaste, tooth powder, toothbrush, etc. Yes purchased or received in-kind over the past 12 months? No

What is the money value of the toothpaste, tooth

powder, toothbrush, etc. purchased or received in-kind by your household during the past 30 days? (Rupees)

What is the money value of the toothpaste, tooth

powder, toothbrush, etc. purchased or received in-kind by your household during the past 12 months? (Rupees)

Were other personal care items (shampoo, combs, Yes

cosmetics, etc.) purchased or received in-kind over No the past 12 months?

What is the money value of the personal care items

(shampoo, combs, cosmetics, etc.) purchased or received in-kind by your household during the past 30

days? (Rupees)

What is the money value of the personal care items

(shampoo, combs, cosmetics, etc.) purchased or received in-kind by your household during the past 12

months? (Rupees)

Were dry cleaning and washing expenses done over the Yes past 12 months? No

What is the amount spent by your household on dry

cleaning and washing expenses during the past 30 days? (Rupees)

What is the amount spent by your household on dry

cleaning and washing expenses during the past 12 months? (Rupees)

Were expenses incurred for personal services (haircut, Yes shaving, shoeshine, etc.) over the past 12 months? No

What is the amount spent by your household on personal

services (haircut, shaving, shoeshine, etc.) during the past 30 days? (Rupees)

What is the amount spent by your household on personal

services (haircut, shaving, shoeshine, etc.) during the past 12 months? (Rupees)

Were expenses incurred for Public transportation Yes

(buses, taxis, rickshaws, train tickets, etc.) over No the past 12 months?

What is the amount spent by your household on Public

transportation (buses, taxis, rickshaws, train tickets, etc.) during the past 30 days? (Rupees)

What is the amount spent by your household on Public

transportation (buses, taxis, rickshaws, train tickets, etc.) during the past 12 months? (Rupees)

Were expenses incurred for Petrol, diesel, motor oil Yes

(for personal vehicle only) over the past 12 months? No

What is the money value of the petrol, diesel, motor

oil (for personal vehicle only) purchased or received in-kind by your household during the past 30 days?

(Rupees)

What is the money value of the petrol, diesel, motor

oil (for personal vehicle only) purchased or received in-kind by your household during the past 12 months?

(Rupees)

Were expenses incurred for entertainment (cinema, Yes

CD/cassette rentals, etc.) over the past 12 months? No

What is the amount spent on entertainment (cinema,

CD/cassette rentals, etc.) by your household during the past 30 days? (Rupees)

What is the amount spent on entertainment (cinema,

CD/cassette rentals, etc.) by your household during the past 12 months? (Rupees)

Were expenses incurred for newspapers, books, Yes stationery supplies (except educational expenses) over No the past 12 months?

What is the amount spent on newspapers, books,

stationery supplies (except educational expenses) by your household during the past 30 days? (Rupees)

What is the amount spent on newspapers, books,

stationery supplies (except educational expenses) by your household during the past 12 months? (Rupees)

Were expenses incurred for pocket money for children Yes over the past 12 months? No

What is the amount spent on pocket money for children

by your household during the past 30 days? (Rupees)

What is the amount spent on pocket money for children

by your household during the past 12 months? (Rupees)

Were expenses incurred for educational and Yes

professional services over the past 12 months? No

What is the amount spent on educational and

professional services by your household during the past 30 days? (Rupees)

What is the amount spent on educational and

professional services by your household during the past 12 months? (Rupees)

Were expenses incurred for modern medicines and health Yes services (doctor fees, hospital charges etc.) over the No past 12 months?

What is the amount spent on modern medicines and

health services (doctor fees, hospital charges etc.) by your household during the past 30 days? (Rupees)

What is the amount spent on modern medicines and

health services (doctor fees, hospital charges etc.) by your household during the past 12 months? (Rupees)

Were expenses incurred for traditional medicines and Yes

health services over the past 12 months? No

What is the amount spent on traditional medicines and

health services by your household during the past 30 days? (Rupees)

What is the amount spent on traditional medicines and

health services by your household during the past 12 months? (Rupees)

Were expenses incurred as wages paid to watchman, Yes

servant, gardener, driver, etc. over the past 12 No months?

What is the amount spent on wages paid to watchman,

servant, gardener, driver, etc. by your household during the past 30 days? (Rupees)

What is the amount spent on wages paid to watchman,

servant, gardener, driver, etc. by your household during the past 12 months? (Rupees)

Were light bulbs, shades, batteries, etc. purchased or Yes

received in-kind over the past 12 months? No

What is the money value of the light bulbs, shades,

batteries, etc. purchased or received in-kind by your household during the past 30 days? (Rupees)

What is the money value of the light bulbs, shades,

batteries, etc. purchased or received in-kind by your household during the past 12 months? (Rupees)

Were household cleaning articles (soap, bleach, Yes

washing powder, etc.) purchased or received in-kind No over the past 12 months?

What is the money value of the household cleaning

articles (soap, bleach, washing powder, etc.) purchased or received in-kind by your household during

the past 30 days? (Rupees)

What is the money value of the household cleaning

articles (soap, bleach, washing powder, etc.) purchased or received in-kind by your household during

the past 12 months? (Rupees)

**INFREQUENT EXPENSES**

Were expenses incurred as legal expenses and insurance Yes (life, car, etc.) over the past 12 months? No

What is the amount spent or received in-kind by your

household on legal expenses and insurance (life, car, etc.) during the past 12 months? (Rupees)

Were expenses incurred as income taxes, land taxes, Yes

housing and property taxes over the past 12 months? No

What is the amount spent or received in-kind by your

household on income taxes, land taxes, housing and property taxes during the past 12 months? (Rupees)

Were expenses incurred on repair and other expenses Yes for personal vehicle (registration, fines) over the No past 12 months?

What is the amount spent or received in-kind by your

household on repair and other expenses for personal vehicle (registration, fines) during the past 12

months? (Rupees)

Were expenses incurred on postal expenses, telegrams, Yes fax, telephone over the past 12 months? No

What is the amount spent or received in-kind by your

household postal expenses, telegrams, fax, telephone during the past 12 months? (Rupees)

Were expenses incurred for excursions, holidays, Yes

(including travel and lodging) over the past 12 No months?

What is the amount spent or received in-kind by your

household on excursions, holidays, (including travel and lodging) during the past 12 months? (Rupees)

Were toys, sports goods purchased or received in-kind Yes over the past 12 months? No

What is the money value of the amount purchased or

received in-kind by your household on toys, sports goods during the past 12 months? (Rupees)

Were expenses incurred for repair and maintenance of Yes the house over the past 12 months? No

What is the amount spent or received in-kind by your

household on repair and maintenance of the house during the past 12 months? (Rupees)

Were expenses incurred for repair and servicing of Yes

household effects over the past 12 months? No

What is the amount spent or received in-kind by your

household on repair and servicing of household effects during the past 12 months? (Rupees)

Were expenses incurred for home improvements and Yes

additions over the past 12 months? No

What is the amount spent or received in-kind by your

household on home improvements and additions during the past 12 months? (Rupees)

Were expenses incurred for marriages, births, and Yes

other ceremonies over the past 12 months? No

What is the amount spent or received in-kind by your

household on marriages, births, and other ceremonies during the past 12 months? (Rupees)

Were expenses incurred for dowry & bride price given Yes

over the past 12 months? No

What is the amount spent or received in-kind by your

household on dowry & bride price during the past 12 months? (Rupees)

Was a dowry & bride price received the past 12 months? Yes

No

What is the amount received by your household for

dowry & bride price during the past 12 months? (Rupees)

Were expenses incurred as funeral and death related Yes

expenses the past 12 months? No

What is the amount spent or received in-kind by your

household on funeral and death related during the past 12 months? (Rupees)

Were expenses incurred as expenditure on religious Yes

ceremonies the past 12 months? No

What is the amount spent or received in-kind by your

household for expenditure on religious ceremonies during the past 12 months? (Rupees)

Were expenses incurred as charity the past 12 months? Yes

No

What is the amount spent by your household on charity

during the past 12 months? (Rupees)

What is the amount received in-kind by your household

on charity during the past 12 months? (Rupees)

Did you incur cash losses in the past 12 months? Yes No

What is the amount lost by your household during the

past 12 months? (Rupees)

Were expenses incurred for gifts and donations the Yes

past 12 months? No

What is the amount spent by your household on gifts

and donations during the past 12 months? (Rupees)

What is the amount received in-kind by your household

in gifts and donations during the past 12 months? (Rupees)

Were crockery, cutlery and kitchen utensils (household Yes use) purchased or received in-kind over the past 12 No months?

What is the money value of the amount purchased or

received in-kind by your household on crockery, cutlery and kitchen utensils (household use) during

the past 12 months? (Rupees)

Were Kitchen appliances (refrigerator, cooking range, Yes

blenders, etc.) purchased or received in-kind over the No past 12 months?

What is the money value of the kitchen appliances

(refrigerator, cooking range, blenders, etc.) purchased or received in-kind by your household

during the past 12 months? (Rupees)

Were pillows, mattresses, blankets, etc. purchased or Yes

received in-kind over the past 12 months? No

What is the money value of the pillows, mattresses,

blankets, etc. purchased or received in-kind by your household during the past 12 months? (Rupees)

Were Jewelry, watches purchased or received in-kind Yes

over the past 12 months? No

What is the money value of the Jewelry, watches

purchased or received in-kind by your household during the past 12 months? (Rupees)

Were furniture and fixtures purchased or received Yes

in-kind over the past 12 months? No

What is the money value of the furniture and fixtures

purchased or received in-kind by your household during the past 12 months? (Rupees)

Were electric fans purchased or received in-kind over Yes

the past 12 months? No

What is the money value of the electric fans purchased

or received in-kind by your household during the past 12 months? (Rupees)

Were heaters (electric, gas, kerosene) purchased or Yes

received in-kind over the past 12 months? No

What is the money value of the heaters (electric, gas,

kerosene) purchased or received in-kind by your household during the past 12 months? (Rupees)

Were sewing machines purchased or received in-kind Yes

over the past 12 months? No

What is the money value of the sewing machines

purchased or received in-kind by your household during the past 12 months? (Rupees)

Was an iron (electric or other) purchased or received Yes

in-kind over the past 12 months? No

What is the money value of the iron (electric or

other) purchased or received in-kind by your household during the past 12 months? (Rupees)

Was a washing machine purchased or received in-kind Yes over the past 12 months? No

What is the money value of the washing machine

purchased or received in-kind by your household during the past 12 months? (Rupees)

Was a cassette recorder or player, radio, etc. Yes purchased or received in-kind over the past 12 months? No

What is the money value of the cassette recorder or

player, radio, etc. purchased or received in-kind by your household during the past 12 months? (Rupees)

Was a camera, camcorder, etc. purchased or received Yes in-kind over the past 12 months? No

What is the money value of the camera, camcorder

purchased or received in-kind by your household during the past 12 months? (Rupees)

Was a bicycle purchased or received in-kind over the Yes

past 12 months? No

What is the money value of the bicycle purchased or

received in-kind by your household during the past 12 months? (Rupees)

Was a motorcycle purchased or received in-kind over Yes

the past 12 months? No

What is the money value of the motorcycle purchased or

received in-kind by your household during the past 12 months? (Rupees)

Was a motor car or other such vehicle purchased or Yes

received in-kind over the past 12 months? No

What is the money value of the motor car or other such

vehicle purchased or received in-kind by your household during the past 12 months? (Rupees)

Were other durable goods (bullock/he buffalo carts, Yes

etc.) purchased or received in-kind over the past 12 No months?

What is the money value of the other durable goods

(bullock/he buffalo carts, etc.) purchased or received in-kind by your household during the past 12 months?

(Rupees)

Were pressure lamps/petromax purchased or received Yes in-kind over the past 12 months? No

What is the money value of the pressure lamps/petromax

purchased or received in-kind by your household during the past 12 months? (Rupees)

Were telephone sets / cordless/mobile phone/pager Yes purchased or received in-kind over the past 12 months? No

What is the money value of the telephone sets /

cordless/mobile phone/pager purchased or received in-kind by your household during the past 12 months?

(Rupees)

Were computer, printers purchased or received in-kind Yes over the past 12 months? No

What is the money value of the computer, printers

purchased or received in-kind by your household during the past 12 months? (Rupees)

**PART:C INVENTORY OF DURABLE GOODS**

Does your household own radio, cassette or CD players? Yes

No

How many radio, cassette or cd player does your

household own?

How many years ago did you acquire radio, cassette or cd player?

(IF MORE THAN ONE ITEM OWNED, ASK ABOUT MOST RECENTLY ACQUIRED ITEM)

Did you purchase it, receive it as a gift or payment PURCHASE

for services, or receive it as dowry or inheritance? GIFT/PAYMENT DOWRY/INHERITANCE

(IF MORE THAN ONE ITEM OWNED, ASK ABOUT MOST RECENTLY ACQUIRED ITEM)

How much was it worth when you acquired it? (In Rupees)

(IF MORE THAN ONE ITEM OWNED, ASK ABOUT MOST RECENTLY ACQUIRED ITEM)

If you wanted to sell this radio, cassette or cd

player today, how much money would you receive for it?

(In Rupees) " (IF MORE THAN ONE ITEM OWNED, ASK ABOUT TOTAL VAL OF ALL ITEMS)

Does your household own camera (still movie)? Yes No

How many camera (still movie) does your household own?

How many years ago did you acquire the camera (still movie)?

(IF MORE THAN ONE ITEM OWNED, ASK ABOUT MOST RECENTLY ACQUIRED ITEM)

Did you purchase it, receive it as a gift or payment PURCHASE

for services, or receive it as dowry or inheritance? GIFT/PAYMENT DOWRY/INHERITANCE

(IF MORE THAN ONE ITEM OWNED, ASK ABOUT MOST RECENTLY ACQUIRED ITEM)

How much was it worth when you acquired it? (In Rupees)

(IF MORE THAN ONE ITEM OWNED, ASK ABOUT MOST RECENTLY ACQUIRED ITEM)

If you wanted to sell this camera (still movie) today,

how much money would you receive for it?(In Rupees) "

(IF MORE THAN ONE ITEM OWNED, ASK ABOUT TOTAL VAL OF ALL ITEMS)

Does your household own bicycle? Yes No

How many bicycle does your household own?

How many years ago did you acquire the bicycle?

(IF MORE THAN ONE ITEM OWNED, ASK ABOUT MOST RECENTLY ACQUIRED ITEM)

Did you purchase it, receive it as a gift or payment PURCHASE

for services, or receive it as dowry or inheritance? GIFT/PAYMENT DOWRY/INHERITANCE

(IF MORE THAN ONE ITEM OWNED, ASK ABOUT MOST RECENTLY ACQUIRED ITEM)

How much was it worth when you acquired it? (In Rupees)

(IF MORE THAN ONE ITEM OWNED, ASK ABOUT MOST RECENTLY ACQUIRED ITEM)

If you wanted to sell this bicycle today, how much

money would you receive for it?(In Rupees)"

(IF MORE THAN ONE ITEM OWNED, ASK ABOUT TOTAL VAL OF ALL ITEMS)

Does your household own motorcycle/scooter? Yes No

How many motorcycle/scooter does your household own?

How many years ago did you acquire motorcycle/scooter?

(IF MORE THAN ONE ITEM OWNED, ASK ABOUT MOST RECENTLY ACQUIRED ITEM)

Did you purchase it, receive it as a gift or payment PURCHASE

for services, or receive it as dowry or inheritance? GIFT/PAYMENT DOWRY/INHERITANCE

(IF MORE THAN ONE ITEM OWNED, ASK ABOUT MOST RECENTLY ACQUIRED ITEM)

How much was it worth when you acquired it? (In Rupees)

(IF MORE THAN ONE ITEM OWNED, ASK ABOUT MOST RECENTLY ACQUIRED ITEM)

If you wanted to sell this motorcycle/scooter today,

how much money would you receive for it?(In Rupees)

(IF MORE THAN ONE ITEM OWNED, ASK ABOUT TOTAL VAL OF ALL ITEMS)

Does your household own motorcar? Yes No

How many motorcar(s) does your household own?

How many years ago did you acquire motorcar?

(IF MORE THAN ONE ITEM OWNED, ASK ABOUT MOST RECENTLY ACQUIRED ITEM)

Did you purchase it, receive it as a gift or payment PURCHASE

for services, or receive it as dowry or inheritance? GIFT/PAYMENT DOWRY/INHERITANCE

(IF MORE THAN ONE ITEM OWNED, ASK ABOUT MOST RECENTLY ACQUIRED ITEM)

How much was it worth when you acquired it? (In Rupees)

(IF MORE THAN ONE ITEM OWNED, ASK ABOUT MOST RECENTLY ACQUIRED ITEM)

If you wanted to sell this motorcar today, how much

money would you receive for it?(In Rupees)

(IF MORE THAN ONE ITEM OWNED, ASK ABOUT TOTAL VAL OF ALL ITEMS)

Does your household own a refrigerator/freeze? Yes No

How many refrigerator/freeze(s) does your household own?

How many years ago did you acquire

refrigerator/freeze?

(IF MORE THAN ONE ITEM OWNED, ASK ABOUT MOST RECENTLY ACQUIRED ITEM)

Did you purchase it, receive it as a gift or payment PURCHASE

for services, or receive it as dowry or inheritance? GIFT/PAYMENT DOWRY/INHERITANCE

(IF MORE THAN ONE ITEM OWNED, ASK ABOUT MOST RECENTLY ACQUIRED ITEM)

How much was it worth when you acquired it? (In Rupees)

(IF MORE THAN ONE ITEM OWNED, ASK ABOUT MOST RECENTLY ACQUIRED ITEM)

If you wanted to sell this refrigerator/freeze today,

how much money would you receive for it?(In Rupees)

(IF MORE THAN ONE ITEM OWNED, ASK ABOUT TOTAL VAL OF ALL ITEMS)

Does your household own washing machine? Yes No

How many washing machine(s) does your household own?

How many years ago did you acquire washing machine?

(IF MORE THAN ONE ITEM OWNED, ASK ABOUT MOST RECENTLY ACQUIRED ITEM)

Did you purchase it, receive it as a gift or payment PURCHASE

for services, or receive it as dowry or inheritance? GIFT/PAYMENT DOWRY/INHERITANCE

(IF MORE THAN ONE ITEM OWNED, ASK ABOUT MOST RECENTLY ACQUIRED ITEM)

How much was it worth when you acquired it? (In Rupees)

(IF MORE THAN ONE ITEM OWNED, ASK ABOUT MOST RECENTLY ACQUIRED ITEM)

If you wanted to sell this washing machine today, how

much money would you receive for it? (In Rupees)

(IF MORE THAN ONE ITEM OWNED, ASK ABOUT TOTAL VAL OF ALL ITEMS)

Does your household own fans? Yes

No

How many fans does your household own?

How many years ago did you acquire fans?

(IF MORE THAN ONE ITEM OWNED, ASK ABOUT MOST RECENTLY ACQUIRED ITEM)

Did you purchase it, receive it as a gift or payment PURCHASE

for services, or receive it as dowry or inheritance? GIFT/PAYMENT DOWRY/INHERITANCE

(IF MORE THAN ONE ITEM OWNED, ASK ABOUT MOST RECENTLY ACQUIRED ITEM)

How much was it worth when you acquired it? (In Rupees)

(IF MORE THAN ONE ITEM OWNED, ASK ABOUT MOST RECENTLY ACQUIRED ITEM)

If you wanted to sell this fans today, how much money

would you receive for it? (In Rupees)

(IF MORE THAN ONE ITEM OWNED, ASK ABOUT TOTAL VAL OF ALL ITEMS)

Does your household own heaters? Yes No

How many heaters does your household own?

How many years ago did you acquire heaters?

(IF MORE THAN ONE ITEM OWNED, ASK ABOUT MOST RECENTLY ACQUIRED ITEM)

Did you purchase it, receive it as a gift or payment PURCHASE

for services, or receive it as dowry or inheritance? GIFT/PAYMENT DOWRY/INHERITANCE

(IF MORE THAN ONE ITEM OWNED, ASK ABOUT MOST RECENTLY ACQUIRED ITEM)

How much was it worth when you acquired it? (In Rupees)

(IF MORE THAN ONE ITEM OWNED, ASK ABOUT MOST RECENTLY ACQUIRED ITEM)

If you wanted to sell this heaters today, how much

money would you receive for it? (In Rupees)

(IF MORE THAN ONE ITEM OWNED, ASK ABOUT TOTAL VAL OF ALL ITEMS)

Does your household own television/VCR/VCD Player? Yes No

How many television/VCR/VCD Player does your household own?

How many years ago did you acquire television/VCR/VCD Player?

(IF MORE THAN ONE ITEM OWNED, ASK ABOUT MOST RECENTLY ACQUIRED ITEM)

Did you purchase it, receive it as a gift or payment PURCHASE

for services, or receive it as dowry or inheritance? GIFT/PAYMENT DOWRY/INHERITANCE

(IF MORE THAN ONE ITEM OWNED, ASK ABOUT MOST RECENTLY ACQUIRED ITEM)

How much was it worth when you acquired it? (In Rupees)

(IF MORE THAN ONE ITEM OWNED, ASK ABOUT MOST RECENTLY ACQUIRED ITEM)

If you wanted to sell this Television/VCR/VCD Player

today, how much money would you receive for it? (In

Rupees) (IF MORE THAN ONE ITEM OWNED, ASK ABOUT TOTAL VAL OF ALL ITEMS)

Does your household own pressure lamps/petromax? Yes No

How many pressure lamps/petromax does your household own?

How many years ago did you acquire pressure

lamps/petromax?

(IF MORE THAN ONE ITEM OWNED, ASK ABOUT MOST RECENTLY ACQUIRED ITEM)

Did you purchase it, receive it as a gift or payment PURCHASE

for services, or receive it as dowry or inheritance? GIFT/PAYMENT DOWRY/INHERITANCE

(IF MORE THAN ONE ITEM OWNED, ASK ABOUT MOST RECENTLY ACQUIRED ITEM)

How much was it worth when you acquired it? (In Rupees)

(IF MORE THAN ONE ITEM OWNED, ASK ABOUT MOST RECENTLY ACQUIRED ITEM)

If you wanted to sell this pressure lamps/petromax

today, how much money would you receive for it? (In

Rupees) (IF MORE THAN ONE ITEM OWNED, ASK ABOUT TOTAL VAL OF ALL ITEMS)

Does your household own telephone sets/cordless/mobile Yes phone/pager? No

How many telephone sets/cordless/mobile phone/pager

does your household own?

How many years ago did you acquire telephone

sets/cordless/mobile phone/pager?

(IF MORE THAN ONE ITEM OWNED, ASK ABOUT MOST RECENTLY ACQUIRED ITEM)

Did you purchase it, receive it as a gift or payment PURCHASE

for services, or receive it as dowry or inheritance? GIFT/PAYMENT DOWRY/INHERITANCE

(IF MORE THAN ONE ITEM OWNED, ASK ABOUT MOST RECENTLY ACQUIRED ITEM)

How much was it worth when you acquired it? (In Rupees)

(IF MORE THAN ONE ITEM OWNED, ASK ABOUT MOST RECENTLY ACQUIRED ITEM)

If you wanted to sell this telephone

sets/cordless/mobile phone/pager today, how much money

would you receive for it? (In Rupees) (IF MORE THAN ONE ITEM OWNED, ASK ABOUT TOTAL VAL OF ALL ITEMS)

Does your household own sewing machines? Yes No

How many sewing machines does your household own?

How many years ago did you acquire sewing machines?

(IF MORE THAN ONE ITEM OWNED, ASK ABOUT MOST RECENTLY ACQUIRED ITEM)

Did you purchase it, receive it as a gift or payment PURCHASE

for services, or receive it as dowry or inheritance? GIFT/PAYMENT DOWRY/INHERITANCE

(IF MORE THAN ONE ITEM OWNED, ASK ABOUT MOST RECENTLY ACQUIRED ITEM)

How much was it worth when you acquired it? (In Rupees)

(IF MORE THAN ONE ITEM OWNED, ASK ABOUT MOST RECENTLY ACQUIRED ITEM)

If you wanted to sell this sewing machines today, how

much money would you receive for it? (In Rupees)

(IF MORE THAN ONE ITEM OWNED, ASK ABOUT TOTAL VAL OF ALL ITEMS)

Does your household own furniture, rugs, clocks? Yes No

How many furniture, rugs, clocks does your household own?

How many years ago did you acquire furniture, rugs, clocks?

(IF MORE THAN ONE ITEM OWNED, ASK ABOUT MOST RECENTLY ACQUIRED ITEM)

Did you purchase it, receive it as a gift or payment PURCHASE

for services, or receive it as dowry or inheritance? GIFT/PAYMENT DOWRY/INHERITANCE

(IF MORE THAN ONE ITEM OWNED, ASK ABOUT MOST RECENTLY ACQUIRED ITEM)

How much was it worth when you acquired it? (In Rupees)

(IF MORE THAN ONE ITEM OWNED, ASK ABOUT MOST RECENTLY ACQUIRED ITEM)

If you wanted to sell this furniture, rugs, clocks

today, how much money would you receive for it? (In

Rupees) (IF MORE THAN ONE ITEM OWNED, ASK ABOUT TOTAL VAL OF ALL ITEMS)

Does your household own kitchen utensils? Yes No

How many kitchen utensils does your household own?

How many years ago did you acquire kitchen utensils?

(IF MORE THAN ONE ITEM OWNED, ASK ABOUT MOST RECENTLY ACQUIRED ITEM)

Did you purchase it, receive it as a gift or payment PURCHASE

for services, or receive it as dowry or inheritance? GIFT/PAYMENT DOWRY/INHERITANCE

(IF MORE THAN ONE ITEM OWNED, ASK ABOUT MOST RECENTLY ACQUIRED ITEM)

How much was it worth when you acquired it? (In Rupees)

(IF MORE THAN ONE ITEM OWNED, ASK ABOUT MOST RECENTLY ACQUIRED ITEM)

If you wanted to sell this kitchen utensils today, how

much money would you receive for it? (In Rupees)

(IF MORE THAN ONE ITEM OWNED, ASK ABOUT TOTAL VAL OF ALL ITEMS)

Does your household own jewelry (including watches)? Yes

No

How many jewelry (including watches) does your

household own?

How many years ago did you acquire jewelry (including watches)?

(IF MORE THAN ONE ITEM OWNED, ASK ABOUT MOST RECENTLY ACQUIRED ITEM)

Did you purchase it, receive it as a gift or payment PURCHASE

for services, or receive it as dowry or inheritance? GIFT/PAYMENT DOWRY/INHERITANCE

(IF MORE THAN ONE ITEM OWNED, ASK ABOUT MOST RECENTLY ACQUIRED ITEM)

How much was it worth when you acquired it? (In Rupees)

(IF MORE THAN ONE ITEM OWNED, ASK ABOUT MOST RECENTLY ACQUIRED ITEM)

If you wanted to sell this jewelry (including watches)

today, how much money would you receive for it? (In

Rupees) (IF MORE THAN ONE ITEM OWNED, ASK ABOUT TOTAL VAL OF ALL ITEMS)

Does your household own computer/printers? Yes No

How many computer/printers does your household own?

How many years ago did you acquire the

computer/printers?

(IF MORE THAN ONE ITEM OWNED, ASK ABOUT MOST RECENTLY ACQUIRED ITEM)

Did you purchase it, receive it as a gift or payment PURCHASE

for services, or receive it as dowry or inheritance? GIFT/PAYMENT DOWRY/INHERITANCE

(IF MORE THAN ONE ITEM OWNED, ASK ABOUT MOST RECENTLY ACQUIRED ITEM)

How much was it worth when you acquired it? (In Rupees)

(IF MORE THAN ONE ITEM OWNED, ASK ABOUT MOST RECENTLY ACQUIRED ITEM)

If you wanted to sell this computer printers today,

how much money would you receive for it? (In Rupees)

(IF MORE THAN ONE ITEM OWNED, ASK ABOUT TOTAL VAL OF ALL ITEMS)

**Part D: Own account production of goods**

Were dalo, nanglo, doko, namlo, rope, mudha, etc. Yes produced and consumed by your household over the past No 12 months?

What is the monetary value in the local market of the

items produced and consumed yourself during the past: 30 days? In rupees

What is the monetary value in the local market of the

items produced and consumed yourself during the past: 12 months? In rupees

Were Mandro, Mat, Sukul, Bhakari, Ghum, Chitro, Broom, Yes etc.produced and consumed by your household over the No past 12 months?

What is the monetary value in the local market of the

items produced and consumed yourself during the past: 30 days? In rupees

What is the monetary value in the local market of the

items produced and consumed yourself during the past: 12 months? In rupees

Were Radi, Pakhi, Homespun clothes, etc. produced and Yes consumed by your household over the past 12 months? No

What is the monetary value in the local market of the

items produced and consumed yourself during the past: 30 days? In rupees

What is the monetary value in the local market of the

items produced and consumed yourself during the past: 12 months? In rupees

Were Firewood/Dung collection produced and consumed by Yes your household over the past 12 months? No

What is the monetary value in the local market of the

items produced and consumed yourself during the past: 30 days? In rupees

What is the monetary value in the local market of the

items produced and consumed yourself during the past: 12 months? In rupees

Were Furniture and allied wooden materials produced Yes

and consumed by your household over the past 12 No months?

What is the monetary value in the local market of the

items produced and consumed yourself during the past: 30 days? In rupees

What is the monetary value in the local market of the

items produced and consumed yourself during the past: 12 months? In rupees

Were Sickle, Chulesi, Knife, etc. produced and Yes consumed by your household over the past 12 months? No

What is the monetary value in the local market of the

items produced and consumed yourself during the past: 30 days? In rupees

What is the monetary value in the local market of the

items produced and consumed yourself during the past: 12 months? In rupees

Were tailoring done for household purposes by your Yes

household over the past 12 months? No

What is the monetary value in the local market of the

tailoring done yourself during the past: 30 days? In rupees

What is the monetary value in the local market of the

tailoring done yourself during the past: 12 months? In rupees

Were Shoe making/repairing done by your household Yes

over the past 12 months? No

What is the monetary value in the local market of the

Shoe making/repairing done by yourself during the past: 30 days? In rupees

What is the monetary value in the local market of the

Shoe making/repairing done by yourself during the past: 12 months? In rupees

Were water fetching done by your household over the Yes past 12 months? No

What is the monetary value in the local market of the

water fetching done by yourself during the past: 30 days? In rupees

What is the monetary value in the local market of the

water fetching done by yourself during the past: 12 months? In rupees

Were minor house repairing done by your household over Yes the past 12 months? No

What is the monetary value in the local market of the

minor house repairing done by yourself during the past: 30 days? In rupees

What is the monetary value in the local market of the

minor house repairing done by yourself during the past: 12 months? In rupees

Were biogas produced and consumed by your household Yes over the past 12 months? No

What is the monetary value in the local market of the

items produced and consumed yourself during the past: 30 days? In rupees

What is the monetary value in the local market of the

items produced and consumed yourself during the past: 12 months? In rupees

Were Pickle, Gundruk, Masyaura, Titaura, Jam, etc. Yes produced and consumed by your household over the past No 12 months?

What is the monetary value in the local market of the

items produced and consumed yourself during the past: 30 days? In rupees

What is the monetary value in the local market of the

items produced and consumed yourself during the past: 12 months? In rupees

Were Other (Communal construction, Duna, Tapari, Yes Batti, etc.) produced and consumed by your household No over the past 12 months?

What is the monetary value in the local market of the

items produced and consumed yourself during the past: 30 days? In rupees

What is the monetary value in the local market of the

items produced and consumed yourself during the past: 12 months? In rupees

**Section 7 - Education**

Do you have children? Yes

No

How many children do you have?

Do your children go to school? Yes

No

How many of them go to school? 1

2

3

4

5

6

7

8

9

10

What type of school/college is ..[CHILD 1].. Currently Community/government attending? Institutional/private

Technical/vocational Gurukul / madarsha / gumba Community (public) campus Private campus

Constituent (angik) campus Other

What type of school/college is ..[CHILD 2].. Currently Community/government attending? Institutional/private

Technical/vocational Gurukul / madarsha / gumba Community (public) campus Private campus

Constituent (angik) campus Other

What type of school/college is ..[CHILD 3].. Currently Community/government attending? Institutional/private

Technical/vocational Gurukul / madarsha / gumba Community (public) campus Private campus

Constituent (angik) campus Other

What type of school/college is ..[CHILD 4].. Currently Community/government attending? Institutional/private

Technical/vocational Gurukul / madarsha / gumba Community (public) campus Private campus

Constituent (angik) campus Other

What type of school/college is ..[CHILD 5].. Currently Community/government attending? Institutional/private

Technical/vocational Gurukul / madarsha / gumba Community (public) campus Private campus

Constituent (angik) campus Other

How much has your household spent during the past 12

months for ..[CHILD 1].. schooling including (Tuition fee Other fee (exams, admission, events, etc),

Uniform, Textbook / Supplies, Transportation, Private tuition, Others ,(snacks, tea, etc), ? IF NOTHING WAS SPENT, WRITE ZERO.

How much has your household spent during the past 12

months for ..[CHILD 2].. schooling including (Tuition fee Other fee (exams, admission, events, etc),

Uniform, Textbook / Supplies, Transportation, Private tuition, Others ,(snacks, tea, etc), ? IF NOTHING WAS SPENT, WRITE ZERO.

How much has your household spent during the past 12

months for ..[CHILD 3].. schooling including (Tuition fee Other fee (exams, admission, events, etc),

Uniform, Textbook / Supplies, Transportation, Private tuition, Others ,(snacks, tea, etc), ? IF NOTHING WAS SPENT, WRITE ZERO.

How much has your household spent during the past 12

months for ..[CHILD 4].. schooling including (Tuition fee Other fee (exams, admission, events, etc),

Uniform, Textbook / Supplies, Transportation, Private tuition, Others ,(snacks, tea, etc), ? IF NOTHING WAS SPENT, WRITE ZERO.

How much has your household spent during the past 12

months for ..[CHILD 5].. schooling including (Tuition fee Other fee (exams, admission, events, etc),

Uniform, Textbook / Supplies, Transportation, Private tuition, Others ,(snacks, tea, etc), ? IF NOTHING WAS SPENT, WRITE ZERO.

Did ..[CHILD 1].. receive a scholarship to help pay Yes

for their educational expenses? No

How much did ..[CHILD 1].. receive over the past 12

months? (In Rupees)

What is the major form of the scholarship did child 1 Tuition / fees receive? Uniforms/ books

Hostel Other

What is the type of the scholarship? Poor and talented Girls

Dalits

Conflict affected Disabled Hiv/aids

Karnali region Other

Did ..[CHILD 2].. receive a scholarship to help pay Yes

for their educational expenses? No

How much did ..[CHILD 2].. receive over the past 12 months? (Rupees)

What is the major form of the scholarship did child 2 Tuition / fees receive? Uniforms/ books

Hostel Other

What is the type of the scholarship did Child 2 Poor and talented

receive? Girls

Dalits

Conflict affected Disabled Hiv/aids

Karnali region Other

Did ..[CHILD 3].. receive a scholarship to help pay Yes

for their educational expenses? No

How much did ..[CHILD 3].. receive over the past 12 months? (rupees)

What is the major form of the scholarship did child 3 Tuition / fees receive? Uniforms/ books

Hostel Other

What is the type of the scholarship did Child 3 Poor and talented

received? Girls

Dalits

Conflict affected Disabled Hiv/aids

Karnali region Other
