# Supplementary material for: Evaluation of a self-help intervention to promote the health and wellbeing of marginalised people including those living with leprosy in Nepal: a prospective, observational, cluster-based, cohort study with controls
Source: BMC Public Health. 2021 May 6;21:873. doi: 10.1186/s12889-021-10847-0 (PMC8101219; doi:10.1186/s12889-021-10847-0)
Supplement: Supplementary file 2 — Additional file 2. Clinical Data Collection Form (Patients at Risk of Ulcer Sub-Group). Description: Standard clinical data collection form used by researchers in Nepal to describe condition of patients’ limbs and eyes. [file 12889_2021_10847_MOESM2_ESM.docx]

**Clinical Data Collection Form (Patients at Risk of Ulcer Sub-Group)**

Participant ID

**Information regarding Leprosy**

Leprosy Diagnosed (Approximate years)

Leprosy treatment completed Ongoing

Completed


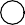

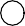

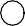


Haven't received treatment yet

Please enter the Released from Treatment date (Approximate years)

Anesthesia of limbs present. Yes

No


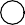

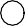


If present, please specify the site Right foot Right hand Left foot Left hand

Duration of anesthesia(approximate months)

**Disability details**

Right Eye Left Eye Right Hand Left Hand Right Foot Left Foot

Absent (WHO grade 0) Sensory loss/impairment

(WHO grade 1)


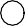

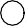


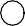

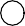

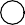

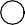

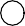

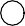

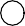

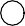

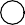

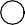


Visible loss/impairment (WHO

grade 2)


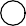


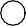

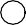

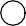

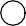

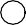


EHF Score

Max disability score

WHO Grading Level of Disability?

**Ulcer Related Information**

Presence of ulcers in hands or foots Yes No


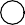

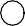


**If Yes, Location of Hand Ulcer**

Palm Dorsum

Right Hand Left Hand

Right palm Thumb

Index finger Middle finger Ring finger Little finger Palm

Right Dorsum Thumb

Index finger Middle finger Ring finger Little finger Back of hand

Left palm Thumb

Index finger Middle finger Ring finger Little finger Palm

Left Dorsum Thumb

Index finger Middle finger Ring finger Little finger Back of hand

**Location of foot Ulcer**

Plantar Dorsum

Right foot Left foot

Right foot plantar Big toe

Long toe Middle toe Ring toe Little toe Forefoot Midfoot Hindfoot/heel

Right foot dorsum Big toe

Long toe Middle toe Ring toe Little toe Forefoot Midfoot Hindfoot

Left foot plantar Big toe

Long toe Middle toe Ring toe Little toe Forefoot Midfoot Hindfoot/heel

Left foot dorsum Big toe

Long toe Middle toe Ring toe Little toe Forefoot Midfoot Hindfoot

Please specify any other site where an ulcer is present

Number of ulcers in hand

Number of ulcers in feet

Duration of largest unhealed ulcer(approximate months)

Length of largest ulcer in cm

Width of ulcer in cm

Depth of ulcer in cm

Ulcer Exudate None (Wound dry)

Scant (tissues moist)


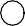

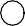

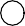


Small (wound wet, moisture evenly distributed, drainage involves 25% or dressing)


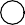
 Moderate (wound tissues saturated, draining may or may not be evenly distributed, drainage involves

25-75% of dressing)


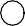
 Copious (wound tissues bathed in fluid, drainage freely expressed)

**Self-Care related Information**

Do you examine your foot regularly? Yes No


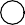

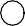


How often do you examine your foot? Daily Weekly Fortnightly

Once in a month Once in a while


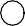

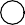

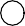

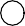

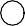

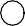

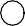


Do you soak your foot regularly in water? Yes No

How often do you soak your foot in water? Daily Weekly Fortnightly

Once in a month Once in a while


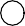

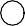

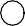

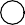

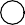


Do you use special footwear? Yes

No


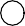

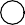


What type of foot wear do you use? Canvas shoes with MCR insole Moulded boot

Sandals with MCR insoles


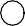

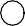

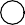

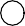

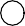

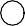


Canvas shoes without MCR insole Normal slipper

Normal leather shoes


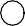

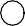


Do you use assistive devices to protect the anesthetic Yes limbs while cooking, farming, using tools? No

What type of assistive device do you use? Please specify

Have you ever attended to any self-help group in the Yes

past? No


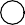

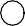


Take photograph of ulcers for PUSH Tool assessment

If more ulcers are present in the different sites please upload the next photo 1.

If the ulcer is present at the different sites please upload the next photo 2.
