# Supplementary material for: Evaluation of a self-help intervention to promote the health and wellbeing of marginalised people including those living with leprosy in Nepal: a prospective, observational, cluster-based, cohort study with controls
Source: BMC Public Health. 2021 May 6;21:873. doi: 10.1186/s12889-021-10847-0 (PMC8101219; doi:10.1186/s12889-021-10847-0)
Supplement: Supplementary file 3 — Additional file 3. Statistical Model. Description: Calculations regarding the precision of the measurement of the effect size of the intervention. [file 12889_2021_10847_MOESM3_ESM.docx]

**Additional File 3: Statistical Model**

One of the four universal outcomes will be monthly household consumption, the analysis of which we describe here.

We have individual $i=1,\ldots,N$ in cluster $j=1,\ldots,J$ at time $t=1,\ldots,T$, where each $t$ represents a month. For each individual, we have (log) monthly households consumption$y_{ijt}$, a set of individual-level (including household) covariates represented by the vector $x_{ijt}$, and cluster-level covariates as the vector $z_{jt}$. Let $d_{ijt}$ be an indicator for whether individual $i$ has the intervention at time $t$. The model specification for analysis then takes the form:

$$\begin{aligned} y_{ijt}=\mu+x_{ijt}^{'}\beta+z_{jt}^{'}\gamma+\delta d_{ijt}+\tau_{t}+\alpha_{ij}+\eta_{jt}+\xi_{j}+e_{ijt}\#\left( 1 \right) \end{aligned}$$

$$\alpha_{ij}\sim N\left( 0,\sigma_{ind|cluster}^{2} \right)$$

$$\eta_{jt}\sim N\left( 0,\sigma_{cluster|time}^{2} \right)$$

$$\xi_{j}\sim N\left( 0,\sigma_{cluster}^{2} \right)$$

$$e_{ijt}\sim N\left( 0,\sigma_{error}^{2} \right)$$

where $\tau_{t}$ are monthly fixed effects (January through December), to capture seasonal effects, and year fixed effects.

*General prior specification*

We specify reasonably informative priors for the model parameters based on our working knowledge of the outcomes and context where possible and weakly informative priors otherwise. These priors are ‘weakly informative’ in the sense of providing only a little information on the location of the parameter while also providing some degree regularisation and computational stability. The prior distributions for the hyperparameters will be common to all models.

We reparameterise the hyperparameters for the specification of our priors. In particular,

$$\begin{aligned} \sigma^{2}=\sigma_{ind|cluster}^{2}+\sigma_{cluster|time}^{2}+\sigma_{cluster}^{2}+\sigma_{error}^{2}\#(1a) \end{aligned}$$

$$ICC=\frac{\sigma_{cluster}^{2}+\sigma_{cluster|time}^{2}}{\sigma^{2}}$$

$$CAC=\frac{\sigma_{cluster}^{2}}{\sigma_{cluster}^{2}+\sigma_{cluster|time}^{2}}$$

$$IAC=\frac{\sigma_{ind|cluster}^{2}}{\sigma_{ind|cluster}^{2}+\sigma_{error}^{2}}$$

where each of these quantities are well-documented in previous studies, particularly cluster randomized trials, and provide a simpler basis for prior specification. $\sigma^{2}$ is the total variance, $ICC$ is the intraclass correlation coefficient (the proportion of variance at the cluster level and hence the correlation between two individuals from the same cluster), $CAC$ is the cluster autocorrelation (the correlation between cluster means between to different points in time), and $IAC$ is the individual autocorrelation (the correlation in individual observations between two points in time). For $\sigma^{2}$ we specify a half-$t_{4}$ prior. The ICC is frequently around 0.05, although values between 0.001 and 0.1 are common, we therefore specify$ICC\sim Beta(2,40)$. Estimates of the $CAC$ and $IAC$ typically vary between 0.8 and 1.0, thus we set a prior of $Beta\left( 18,2 \right)$ for both.

We note that the interpretation of the model parameters in the log-linear model above is that $\exp\left( \delta\right)$, say, is the proportionate increase in consumption associated with a unit change in $d_{ijt}$, i.e. due to the intervention. Our prior for $\delta$, the intervention effect, is that there is only a 5% probability that the intervention has an effect greater than doubling or halving consumption, $\delta\sim N\left( {0,0.35}^{2} \right)$. No similar intervention we are aware of has demonstrated an effect larger than this. Where individual and cluster-level covariates are continuous they will be mean centred and standardized. Based on similar logic we specify $N\left( {0,0.5}^{2} \right)$ priors for all $\beta$ and $\gamma$ parameters as there is little to no evidence to support effect sizes outside this range for any characteristic of rural village dwellers. The Nepal Living Standards Survey 2010/11 estimated the mean total consumption expenditure to be approximately NR 34,000 ranging from NR 13,000 to NR 79,000 for the poorest and richest 20% of the population respectively. We expect our population to be among the poorer part of the population (since the intervention is targeted at them). We therefore specify $\mu\sim N\left( {9,2}^{2} \right)$.

A Bayesian model is defined by a likelihood and a prior. The data from the collected in the study is $Y$. If $\Theta$ is all of the model parameters then the posterior distribution is

$$f\left( \Theta| Y \right)\propto f\left( Y | \Theta\right)f\left( \Theta\right)$$

Interest is in inferences about the treatment effect $\delta$. Our design analysis here is “pre-data” to determine the inferences we would expect to make about the parameters. Data are simulated from model and prior distributions and then used to update the model to obtain the “pre-posterior” distributions of the parameters.

There are different ways of considering the precision afforded by different samples and designs in a Bayesian context. Here we present the average posterior variance:

$$APV=E\left[ Var\left( \delta| Y \right) \right]$$

To give some intuition the design means in terms of the precision of inferences we can consider the size of posterior credible intervals. In particular, we look at the average length criterion (ALC), which asks what the average length of the posterior $\alpha\%$ credible interval is. The length of a posterior credible interval is:

$$l=F_{\delta|Y}^{-1}\left( 1-\frac{\alpha}{2} \right)\boldsymbol{-}F_{\delta|Y}^{-1}\left( \frac{\alpha}{2} \right)$$

and the ALC is:

$$ALC=E\left( l \right)$$

We can also determine the length $l_{0}$ that we have a probability $p$ of observing or smaller:

$$p=\Pr\left( l<l_{0} \right)$$

We simulate data from the model specified above and prior distributions.

The APV for the SHERPA study design is 0.115, and the average length of the 95% credible interval is 0.23. There is an 80% probability that the 95% credible interval will be smaller than 0.17. The mean is greater than the 80% probability length due to the significant right skew: there is a 3% probability that the 95% credible interval will be larger than 1.00. In translate these figures one can consider the smallest effect size for which a 95% credible interval of a given length will exclude zero. For 0.17, this is an effect of 0.085 on the log scale, or an approximate 10% increase in overall monthly household consumption.
